# Intrapancreatic injection of human bone marrow-derived mesenchymal stem/stromal cells alleviates hyperglycemia and modulates the macrophage state in streptozotocin-induced type 1 diabetic mice

**DOI:** 10.1371/journal.pone.0186637

**Published:** 2017-10-26

**Authors:** Norimitsu Murai, Hirokazu Ohtaki, Jun Watanabe, Zhifang Xu, Shun Sasaki, Kazumichi Yagura, Seiji Shioda, Shoichiro Nagasaka, Kazuho Honda, Masahiko Izumizaki

**Affiliations:** 1 Department of Physiology, Showa University School of Medicine, Tokyo, Japan; 2 Department of Anatomy, Showa University School of Medicine, Tokyo, Japan; 3 Division of Diabetes, Metabolism and Endocrinology, Showa University Fujigaoka Hospital, Yokohama, Japan; 4 Center for Biotechnology, Showa University, Tokyo, Japan; 5 Peptide Drug Innovation, Global Research Center for Innovative Life Science, Hoshi University School of Pharmacy and Pharmaceutical Sciences, Tokyo, Japan; Centro Cardiologico Monzino, ITALY

## Abstract

Type 1 diabetes mellitus is a progressive disease caused by the destruction of pancreatic β-cells, resulting in insulin dependency and hyperglycemia. While transplanted bone marrow-derived mesenchymal stem/stromal cells (BMMSCs) have been explored as an alternative therapeutic approach for diseases, the choice of delivery route may be a critical factor determining their sustainability. This study evaluated the effects of intrapancreatic and intravenous injection of human BMMSCs (hBMMSCs) in streptozotocin (STZ)-induced type 1 diabetic mouse model. C57/BL6 mice were intraperitoneally injected with 115 mg/kg STZ on day 0. hBMMSCs (1 × 10^6^ cells) or vehicle were injected into the pancreas or jugular vein on day 7. Intrapancreatic, but not intravenous, hBMMSC injection significantly reduced blood glucose levels on day 28 compared with vehicle injection by the same route. This glucose-lowering effect was not induced by intrapancreatic injection of human fibroblasts as the xenograft control. Intrapancreatically injected fluorescence-labeled hBMMSCs were observed in the intra- and extra-lobular spaces of the pancreas, and intravenously injected cells were in the lung region, although the number of cells mostly decreased within 2 weeks of injection. For hBMMSCs injected twice into the pancreatic region on days 7 and 28, the injected mice had further reduced blood glucose to borderline diabetic levels on day 56. Animals injected with hBMMSCs twice exhibited increases in the plasma insulin level, number and size of islets, insulin-positive proportion of the total pancreas area, and intensity of insulin staining compared with vehicle-injected animals. We found a decrease of Iba1-positive cells in islets and an increase of CD206-positive cells in both the endocrine and exocrine pancreas. The hBMMSC injection also reduced the number of CD40-positive cells merged with glucagon immunoreactions in the islets. These results suggest that intrapancreatic injection may be a better delivery route of hBMMSCs for the treatment of type 1 diabetes mellitus.

## Introduction

Type 1 diabetes mellitus is a progressive disease caused by the destruction of pancreatic β-cells, resulting in insulin dependency and hyperglycemia. Patients with the disease, who require exogenous insulin treatment, are also at risk of blood glucose fluctuations and hypoglycemia. Pancreas or pancreatic islet transplantation is a promising treatment for patients who lack endogenous insulin secretion, particularly those who experience recurrent episodes of severe hypoglycemia [1, 2]. However, the application of this approach is limited by a shortage of organ donors and the need for lifelong administration of immunosuppressive agents, which has potentially adverse effects [3–5].

Stem cell-based therapies have been explored as an alternative therapeutic approach for type 1 diabetes mellitus because of the relatively high availability of stem cells [6]. Among the various types of stem cells, mesenchymal stem/stromal cells (MSCs) are multipotent cells that have the ability to differentiate into cells of mesodermal lineages, and their transplantation carries a low risk of tumorigenesis and few ethical limitations [6–8]. MSCs can be isolated and expanded with high efficiency from several adult and fetal tissues including bone marrow, adipose tissue, dental pulp, and umbilical cord blood [9, 10]. MSCs have regenerative and immunomodulatory properties after their transplantation into human patients and animal models of diseases such as graft versus host disease, heart disease, Crohn’s disease, and stroke [11, 12]. Currently, MSCs derived from human bone marrow (hBMMSCs) represent the most frequently used type of MSC in clinical regenerative medicine [13, 14]. Carlsson et al. have recently shown that hBMMSC transplantation preserves β-cell functions in patients with type 1 diabetes mellitus [15]. The cells were administered only once, intravenously, to these patients. This treatment failed to decrease glycosylated hemoglobin concentrations, but prevented the decreased C-peptide response to a mixed meal tolerance test at the 1 year follow-up evaluation. Further improvements in treatment outcomes might be facilitated by a better understanding of how hBMMSCs function and how they can be applied to treatment of type 1 diabetes mellitus.

Previous studies using animal models have revealed the importance of the delivery route for MSC transplantation efficacy [16, 17]. In animal models, MSCs have been administered intravenously. However, when MSCs were injected intravenously, most cells were trapped in the lungs before reaching target tissues [18–21]. Intravenous hBMMSC transplantation has also been evaluated in mouse models of type 1 diabetes mellitus in preparation for its clinical application [22, 23]. Among such studies, Ho et al. demonstrated that a single intravenous hBMMSC transplantation lowered blood glucose levels, but multiple transplantations at 2 week intervals were necessary to ensure the continuous maintenance of glucose homeostasis [22]. Intra-arterial and local injections of MSCs, which could avoid lung entrapment of MSCs, have been examined in animal models of diseases other than type 1 diabetes mellitus, compared with their systemic injection [24–26]. In rats with traumatic brain injury, intra-arterial transplantation of hBMMSCs leads to higher engraftment levels in the brain than intravenous transplantation [24]. Furthermore, the advantage of local delivery of hBMMSCs into affected joints compared with systemic delivery has been reported in rats with collagen-induced arthritis [25]. These reports suggest that better delivery of the cells to target organs could provide better therapeutic efficacy. We therefore investigated whether the effects of hBMMSCs on glucose homeostasis depend on the selected delivery route of intrapancreatic (local) or intravenous (systemic) injection in an animal model of type 1 diabetes mellitus

Interestingly, in most experimental situations, MSCs exert therapeutic effects without evidence of long-term engraftment because most transplanted cells disappear in a short time [18, 27, 28]. Recently, it has been demonstrated that MSCs play an important role as mediators of inflammatory regulation [11, 29]. Accordingly, their beneficial effects are partly explained by paracrine secretions or cell-to-cell contacts that have multiple effects including crosstalk between MSCs and macrophages [28, 30–33]. Macrophages are classified into two major types: pro-inflammatory (M1) and anti-inflammatory (M2) macrophages. M2 macrophages are further classified into two subtypes: deactivated macrophages, which are induced by IL-10, and alternatively activated macrophages that are induced by IL-4 [34, 35]. We previously reported that local administration of hBMMSCs restores neural functions, increases alternatively activated M2 macrophages in the hippocampus and spinal cord of mouse models of neural damage [27, 36], and decreases the number of M1 macrophages *in vitro* [37]. We suggest that hBMMSCs facilitate restoration of neural functions by modulating the balance between M1 and M2 macrophages.

Here, in the diabetic model induced by streptozotocin (STZ), we revealed that intrapancreatically hBMMSC-injected mice have greater improvements in glucose homeostasis and body weights than intravenously hBMMSC-injected mice, along with cell retention in the pancreas. Intrapancreatically hBMMSC-injected mice have increased plasma insulin levels and improved pancreatic islet histomorphology, suggesting that the injected cells might modify the pancreatic macrophage state and decrease CD40-positive cells in islets.

## Materials and methods

### Animals

Male 7–9-week-old C57/BL6 mice were purchased from Sankyo Lab Service (Tokyo, Japan) and housed at constant temperature and humidity with a 12:12 hour light:dark cycle and *ad libitum* access to a standard diet and water. All experimental procedures involving animals were approved by the Institutional Animal Care and Use Committee of Showa University (#04085, 05116, and 06012).

### Measurement of blood glucose concentrations

Blood samples were obtained from the right facial vein of mice using an animal lancet (Goldenrod, MEDIpoint, Mineola, NY). Blood glucose concentrations were measured with a mini-glucometer (MEDISAFE, Terumo, Tokyo, Japan) capable of measuring glucose concentrations up to 600 mg/dL.

### Preparation of hBMMSCs and adult human dermal fibroblasts (HDFs) for injection

Frozen vials of human MSCs from adult bone marrow (hBMMSCs) were obtained from Dr. Darwin J. Prockop of the Center for the Preparation and Distribution of Adult Stem Cells (http://medicine.tamhsc.edu/irm/msc-distribution.html). This center functions under the auspices of a National Institutes of Health (NIH)/National Center for Research Resources grant (P40 RR 17447–06) and supplies standardized preparations of hBMMSCs enriched for early progenitor cells. All experiments were performed with hBMMSCs from donor 281L. To expand hBMMSCs, a frozen vial of 1 × 10^6^ passage 3 cells was thawed, and the cells were cultured in 20% complete culture medium (CCM) consisting of α-minimum essential medium (Invitrogen, Grand Island, NY) supplemented with 20% heat-inactivated fetal bovine serum (FBS; HyClone, Thermo Fisher Scientific, Waltham, MA), 100 U/mL penicillin and 100 μg/mL streptomycin (Pen/Str, Invitrogen), and 2 mM L-glutamine (Invitrogen). hBMMSCs were seeded at 100 cells/cm^2^ in 20% CCM and incubated at 37°C in a humidified 5% CO_2_ atmosphere. The medium was changed every 3–4 days, and cells were grown to 70–80% confluency (approximately 8 days) [28]. The epitope profile of the cells showed that they were mostly positive for CD29 (>92%), CD44 (>93%), CD90 (>99%), and CD105 (>99%), and negative for CD34 (<1.5%) and CD117 (<0.5%). A sample of hBMMSCs was labeled with fluorescent dyes NIR815 (Cell Vue NIR815 Cell Labeling Kit; Thermo Fisher Scientific, Waltham, MA) or PKH26 (PKH26 Red Fluorescent Cell Linker Kit; Sigma, St Louis, MO), as described previously [36].

HDFs isolated from adult human skin were human dermal fibroblasts and were obtained from ScienCell Research Laboratories (Carlsbad, CA) and cultured in 10% Dulbecco’s modified Eagle’s medium (Invitrogen) with 10% FBS, Pen/Str, and 2 mM L-glutamine at 37°C in a humidified atmosphere with 5% CO_2_ [37].

hBMMSCs and HDFs were harvested with 0.25% trypsin and 1 mM EDTA (Invitrogen), and resuspended in sterile Hank’s balanced salt solution (HBSS, Invitrogen) at 1 × 10^6^ cells/20 μL for intrapancreatic injection or 1 × 10^6^ cells/150 μL for intravenous injection.

### Induction of the diabetic model by STZ injection

Mice were fasted for 4 hours before STZ injection and then administered STZ (115 mg/kg, Wako, Osaka, Japan) in saline by intraperitoneal injection on day 0. Blood glucose concentrations and body weights were measured four times prior to the cell injection to confirm the basal levels of glucose and development of STZ-induced diabetes mellitus: 7 and 3 days before, and 5 and 7 days after STZ injection. Blood was sampled from the right facial vein between 9:00 am and 11:00 am. Drinking water was replaced with a 10% glucose solution (Otsuka, Tokyo, Japan) overnight to avoid hypoglycemia in mice the day after STZ injection. Before cell transplantation, mice with blood glucose concentrations between 220 and 500 mg/dL on days 5 and 7 were considered to have STZ-induced diabetes mellitus.

### Cell transplantation

Diabetic mice were administered 3.5% sevoflurane in N_2_O/O_2_ (2:1) by inhalation for anesthesia. For cell transplantation into the pancreatic region (intrapancreatic injection), the animals were placed in the right lateral position, and an approximately 0.5 cm cut was made in the skin and retroperitoneum to visually confirm the spleen. Twenty microliters of cell suspension (1×10^6^ cells) or vehicle were slowly injected into the pancreatic region adjacent to the spleen, which consists of the tail of the pancreas, using an insulin syringe with a 29-G needle (Low dose; BD Bioscience, San Jose, CA). Then, the animals were sutured to seal their skin and retroperitoneum, and they recovered from the anesthesia. For intravenous injection, the animals were placed in the supine position under anesthesia, and their skin and fascia on the right clavicle were incised. The right jugular vein was exposed, and 150 μL of cell suspension (1×10^6^ cells) or vehicle was slowly injected into the vein (intravenous injection). The skin of the animals was sutured, and they recovered from the anesthesia.

### Immunostaining

All primary and secondary antibodies used in the present study are listed in Table 1. Mice were anesthetized by intraperitoneal injection of 50 mg/kg sodium pentobarbital and perfusion-fixed with saline followed by 4% paraformaldehyde (PFA). The pancreas was then removed and frozen sections were prepared (10 or 20 μm in thickness). The sections were washed with phosphate buffered saline (PBS) and immersed in 0.3% H_2_O_2_ for 30 minutes to quench endogenous peroxidases. The sections were then blocked by incubation with 2.5% normal horse serum (NHS; Vector Laboratories, Burlingame, CA) in PBS and incubated with primary antibodies against insulin, Iba-1, CD206, or CD40 overnight at 4°C. After washing with PBS, the sections were incubated with appropriate biotinylated secondary antibodies for 2 hours. Binding was visualized using an avidin-biotin complex (ABC) solution (Vector Laboratories). For multiple staining, the pancreatic sections were blocked with 5% NHS and incubated with several primary antibodies overnight at 4°C. The sections were washed, incubated with appropriate fluorescence-labeled secondary antibodies for 2 hours, and then with 4,6-diamidine-2-phenylindole dihydrochloride (DAPI, 1:10,000; Roche, Manheim, Germany) to identify cell nuclei.

**Table 1:** Primary and secondary antibodies used for immunohistochemistry.

**Primary**	**Host**	**Clone #**	**Company**	**Catalog #**	**Dilution (fold)**
α-amylase	Rabbit		Sigma Aldrich (St. Louis, MO)	A8273	200
CD40	Armenian hamster (IgM)	HM40-3	eBioscience (San Diego, CA)	14–0402	2,000
CD206	Rat	MR5D3	AbD Serotec (Raleigh, NC)	MCA2235GA	500
F4/80	Rat	CI:A3-1	BMA Biomedicals (Augst, Switzerland)	T-2008	250
Glucagon	Rabbit		Sigma Aldrich	SAB4501137	200
Iba-1	Rabbit		Wako (Osaka, Japan)	019–19741	500
Insulin	Guinea pig		Dako (Glostrup, Denmark)	N1542	100
MHC class II	Rat	M5/114.15.2	eBioscience	14–5321	200
**Secondary**	**Host**	**Conjugation**	**Company**	**Catalog #**	**Dilution (fold)**
Armenian hamster IgM	Mouse	Biotin	BD Biosciences (Franklin Lakes, NJ)	554035	200
		FITC		554033	200
Guinea pig IgG	Goat	Biotin	Vector Laboratories (Burlingame, CA)	BA-7000	200
		Alexa488	Thermo Fisher Scientific (Waltham, MA)	A11073	500
		Alexa546		A11074	
		Alexa647		A21450	
Rabbit IgG	Goat	Biotin	Vector Laboratories	BA-1000	200
		Alexa488	Thermo Fisher Scientific	A11034	500
		Alexa546		A11035	
		Alexa647		A21245	
Rat IgG	Goat	Texas red	Jackson Laboratories (West Grove, PA)	112-075-167	200
		FITC		112-095-167	

CD40, cluster of differentiation 40; CD206, cluster of differentiation 206; FITC, fluorescein isothiocyanate; Iba1, ionized calcium-binding adapter molecule 1; IgG, immunoglobulin G; IgM, immunoglobulin M; MHC, major histocompatibility complex

We checked the specificity of the primary antibodies, using isotype controls. Isotype controls are listed in following Table 2. Mice were anesthetized by intraperitoneal injection of 50 mg/kg sodium pentobarbital and perfusion-fixed with saline followed by 4% PFA. The pancreas was then removed and frozen sections were prepared (10 μm in thickness). The sections were washed with PBS and immersed in 0.3% H_2_O_2_ for 30 minutes to quench endogenous peroxidases. The sections were then blocked by incubation with 2.5% NHS in PBS and incubated with primary antibodies or isotype controls overnight at 4°C. The immunoglobulin content of the isotype control except guinea pig immunoglobulin G (IgG) was equal to that of the primary antibody which is the same host. Immunoglobulin concentration of anti-insulin antibody used in this study was unavailable. The dilution rate of guinea pig IgG was equal to that of anti-insulin antibody. After washing with PBS, the sections were incubated with appropriate biotinylated secondary antibodies for 2 hours. Binding was visualized using an ABC solution. Antibody specificity was confirmed by incubation of tissues in the absence of primary antibodies and the presence of isotype controls (S1 Fig).

**Table 2:** Isotype controls used for immunohistochemistry.

Isotype control	Host	Clone #	Company	Catalog #
Rabbit IgG	Rabbit		Wako	148–09551
Armenian hamster IgM	Armenian hamster (IgM)	HTK204	Bio Legend (San Diego, CA)	401005
Rat IgG	Rat		Wako	147–09521
Guinea pig IgG	Guinea pig		Medical Biological Laboratories (Nagoya, Japan)	PM067

IgG, immunoglobulin G; IgM, immunoglobulin M

### Experiment 1. Comparison of cell delivery routes in terms of blood glucose and body weight

The choice of delivery route may be a critical factor determining the sustainability of implanted MSCs. We first compared the effect of intrapancreatic and/or intravenous injections of hBMMSCs on blood glucose and body weight. On day 7 after STZ injection, the diabetic mice (*n* = 49) were divided into four experimental groups, vehicle-vein group (*n* = 13), hBMMSC-vein group (*n* = 9), vehicle-pancreas group (*n* = 13), and hBMMSC-pancreas group (*n* = 14), and administered the vehicle (HBSS) or a hBMMSC suspension intravenously into the jugular vein (150 μL) or pancreatic region (20 μL) under 3.5% sevoflurane inhalation. The blood glucose concentration and body weight of each mouse were measured on days 14, 21, and 28.

### Experiment 2. Effect of xenografts on blood glucose and body weight

In the present study, we injected human cells into mice. To rule out the xenograft as the cause of glucose homeostasis, we compared the effects of intrapancreatic injections of hBMMSCs and HDFs as the xenograft control. On day 7 after STZ injection, the mice (*n* = 36) were divided into three groups, vehicle-pancreas group (*n* = 11), hBMMSC-pancreas group (*n* = 11), and HDF-pancreas group (*n* = 14), and administered the vehicle (HBSS), a hBMMSC suspension, or a HDF suspension into the pancreatic region (20 μL) under sevoflurane anesthesia. The blood glucose concentration and body weight of each mouse were measured on days 14, 21, and 28.

### Experiment 3. Body and tissue distributions of hBMMSCs

In experiment 3, to compare the body distribution and fate of hBMMSCs resulting from intrapancreatic or intravenous injection into diabetic mice, we performed time-course *in vivo* fluorescence imaging using NIR815-labeled hBMMSCs. We then examined the pancreatic tissue distribution of cells using PKH26-labeled hBMMSCs with intrapancreatic injection.

#### Body distribution of hBMMSCs analyzed by *in vivo* fluorescence imaging

NIR815 is a membrane-binding fluorescent dye with excitation (Ex) at 633 nm and emission (Em) at 776 nm, which can be detected by an *in vivo* fluorescence imager (Clairvivo OPT; Shimadzu, Kyoto, Japan). On day 6 after STZ injection, hair on the body trunk and forelimbs of mice was removed using a depilatory cream (Epilat; Kracie, Tokyo, Japan). The next day, diabetic mice (*n* = 24) were divided into four experimental groups, vehicle-vein group (*n* = 5), hBMMSC-vein group (*n* = 8), vehicle-pancreas group (*n* = 3), and hBMMSC-pancreas group (*n* = 8), and administered the vehicle (HBSS) or a NIR815-labeled hBMMSC suspension (1×10^6^ cells) into the jugular vein (150 μL) or pancreatic region (20 μL) under anesthesia induced by inhalation of 2% isoflurane (Wako, Tokyo, Japan). Immediately after injection, the mice were placed in the supine position on the stage of a fluorescence imager, and fluorescence intensities were measured with an exposure time of 120 seconds. The same measurements were repeatedly performed on days 8, 11, 14, 21, and 28 (1, 4, 7, 14, and 21 days after injection), and total fluorescence intensities were calculated in the left lateral abdomen under the subphrenic area, corresponding to the pancreas, and in the right lateral supraphrenic area corresponding to the right lung. Finally, the intensities were expressed after subtracting the mean fluorescence intensities in mice injected with the vehicle via the same route. The right lung was selected for analysis because fluorescence from the pancreatic area interfered with fluorescence from the left lung in hBMMSC-pancreas animals.

#### Tissue distribution of hBMMSCs in intrapancreatic injection determined by histology

The tissue distribution of hBMMSCs in the pancreas was examined by injecting PKH26-labeled hBMMSCs into the pancreatic region. PKH26 is a membrane-binding fluorescent dye with Ex 551 and Em 567. The labeled cells were then detected by fluorescence microscopy. On day 7 after STZ injection, diabetic mice (*n* = 9) were administered a PKH26-labeled hBMMSC suspension (1×10^6^ cells) directly into the pancreas (20 μL) under sevoflurane anesthesia. On days 14, 21, and 28, the mice (*n* = 3 per day) were anesthetized by intraperitoneal injection of 50 mg/kg sodium pentobarbital and perfusion-fixed with 0.9% saline followed by 4% PFA. Frozen sections (20 μm in thickness) were incubated with a primary antibody against α-amylase, an exocrine pancreas marker, and DAPI (see Table 1). The stained sections were then observed by confocal fluorescence microscopy (A1; Nikon, Tokyo, Japan).

### Experiment 4. Effect of repeated hBMMSC injections

To further determine the effect of repeated intrapancreatic injections of hBMMSCs on blood glucose, body weight, pancreatic histopathology, and plasma insulin levels, we injected hBMMSCs twice into the pancreatic region on days 7 and 28.

On day 7 after STZ injection, diabetic mice (*n* = 23) were divided into two groups and administered the vehicle (HBSS, *n* = 12) or hBMMSCs (*n* = 11) into the pancreatic region (20 μL) under sevoflurane anesthesia. On day 28, both groups were again administered the same treatment received on day 7. Blood glucose concentrations and body weights were measured on days 10, 14, 21, 28, 35, 42, 49, and 56.

#### Histological evaluation of the pancreas

On day 56, the animals (vehicle-pancreas group, *n* = 5; hBMMSC-pancreas group, *n* = 5) were anesthetized with 50 mg/kg intraperitoneal sodium pentobarbital and then perfused transcardially with 0.9% saline followed by 4% PFA. Frozen pancreas sections were prepared at 10 μm in thickness. Pancreas sections were also obtained from non-STZ-treated normal mice (untreated mice; *n* = 3) and at 7 days after STZ injection (without hBMMSC injection; *n* = 4) by the same procedures.

Three sections from each mouse at 800 μm intervals were stained with hematoxylin and eosin (H.E.) and photographed whole as an overview using a digital camera with macro mode (Ricoh CX4, Tokyo, Japan). The adjacent sections were stained with an anti-insulin primary antibody as described below. Based on these sections, we determined the islet area, percentage of the insulin-positive area, and islet number per area using Scion image software (NIH). In brief, edges of the pancreas in the overview image were traced manually, and the total pancreatic area was calculated. The endocrine pancreas (i.e. the total area of insulin-positive clusters) in each section was measured under an AX70 microscope (Olympus, Tokyo, Japan) at 20× magnification with a DP2-BSW imager (Olympus), and the area of each pancreas was determined manually by circling each insulin-positive cluster. The insulin-positive area was automatically calculated by binarizing the dark brown insulin-like immunoreactions in each endocrine pancreas. The insulin-positive proportion of the total pancreas area in the sections was calculated.

To determine the number of sections for histological evaluation, serial sections of the entire pancreas from three untreated mice were prepared at intervals of 200 μm (nine sections). The sections were stained with H.E. and an anti-insulin primary antibody. The area under the curve (AUC) of the number of islets per pancreas area was estimated and compared between different sectioning intervals: 200 (nine sections), 400 (five sections), and 800 (three sections) μm. There were no differences in the AUCs among the intervals (S2 Fig). Therefore, we used three sections at 800 μm intervals for histological evaluation.

#### Plasma insulin enzyme-linked immunosorbent assay (ELISA)

On day 56, blood samples of the animals (vehicle-pancreas group, *n* = 8; hBMMSC-pancreas group, *n* = 8) were collected from the right ventricle before perfusion fixation, and a heparinized plasma sample was obtained to measure the plasma insulin concentration. Blood samples were also collected from non-STZ-injected normal mice (*n* = 4) and on day 7 after STZ injection (without hBMMSC injection; *n* = 3). Plasma insulin concentrations were determined using a mouse insulin ELISA kit (Morinaga, Tokyo, Japan) according to the manufacturer’s instructions.

### Experiment 5. Effect of hBMMSCs on macrophage markers

We have previously reported that hBMMSC injection into parenchyma of the brain and spine improves injury and modifies the type of activated macrophage during ischemia and trauma [27, 36, 37]. Therefore, we examined the effect of macrophage activation and phenotypes on glucose homeostasis to investigate the possible mechanism of hBMMSCs.

On day 7 after STZ injection, diabetic mice (*n* = 12) were administered the vehicle (HBSS, *n* = 6) or hBMMSCs (1×10^6^ cells, *n* = 6) into the pancreatic region under sevoflurane anesthesia and then sacrificed by 4% PFA perfusion fixation on day 28. Frozen pancreas sections were prepared at 10 μm thicknesses for immunohistochemistry of macrophage markers. The sections were stained with anti-Iba1, anti-CD206, and anti-CD40 antibodies (see Table 1) with colorimetric staining by the ABC system. Then, the number of positive cells was counted in islets and exocrine areas (*n* = 5/animal), and the positive cell number in a certain islet or exocrine pancreas area was calculated by Scion image software. Simultaneously, we analyzed pancreatic sections from non-STZ-injected normal mice (*n* = 3). The sections were observed and analyzed under the AX70 microscope at 20× magnification with the DP2-BSW imager.

Iba1- or CD40-positive cells were determined by multiple immunostaining for insulin (for detection of islet), macrophage polarization markers, and glucagon. The sections were assessed by visualization with the A1 confocal fluorescence microscopy system.

### Statistical analysis

Data are expressed as the mean ± standard error of the mean (SEM). Statistical comparisons were conducted using the Student’s t-test for two groups, and one-way analysis of variance with non-parametric multiple comparisons for three or more groups, as indicated in each figure legend. A value of *P* < 0.05 was considered statistically significant.

## Results

### Determination of the optimal STZ dose and definition of the diabetic model

We used mice with STZ-induced type 1 diabetes mellitus in the present study. However, the optimal dose of STZ is quite different according to species and animal suppliers. Therefore, we first determined the optimal STZ dose in C57/BL6 mice obtained from Sankyo Lab. We initially administered saline and STZ at doses of 100 (*n* = 3), 150 (*n* = 3), or 200 mg/kg body weight (*n* = 3) to 8–10-week-old mice via peritoneal injection (200 μL). The mean blood glucose concentration of saline-injected mice was 172 ± 5 mg/dL (mean ± SEM, *n* = 7). All blood glucose levels in 150 mg/kg or 200 mg/kg STZ-treated mice exceeded >600 mg/dL on day 5 whereas the levels in 100 mg/kg STZ-injected animals were no different from those in saline-treated mice. Then, another group of C57BL/6 mice was administered STZ at doses of 115 (*n* = 8) or 130 mg/kg (*n* = 8), and blood glucose concentrations were determined on day 7. While six of eight animals administered STZ at 130 mg/kg exceeded >600 mg/dL, animals injected with 115 mg/kg STZ had mean blood glucose concentrations of 360 ± 46 and 390 ± 43 mg/dL (mean ± SEM, *n* = 8) on days 5 and 7, respectively, and only one animal exceeded >600 mg/dL. Therefore, we determined that 115 mg/kg STZ was optimal for our study. We finally decided to use diabetic mice with a blood glucose range from 220 to 500 mg/dL on days 5 and 7 for subsequent experiments.

### Experiment 1

#### Effect of intrapancreatic and intravenous hBMMSC injections on blood glucose and body weight in STZ-induced type 1 diabetic mice

**Fig 1:**
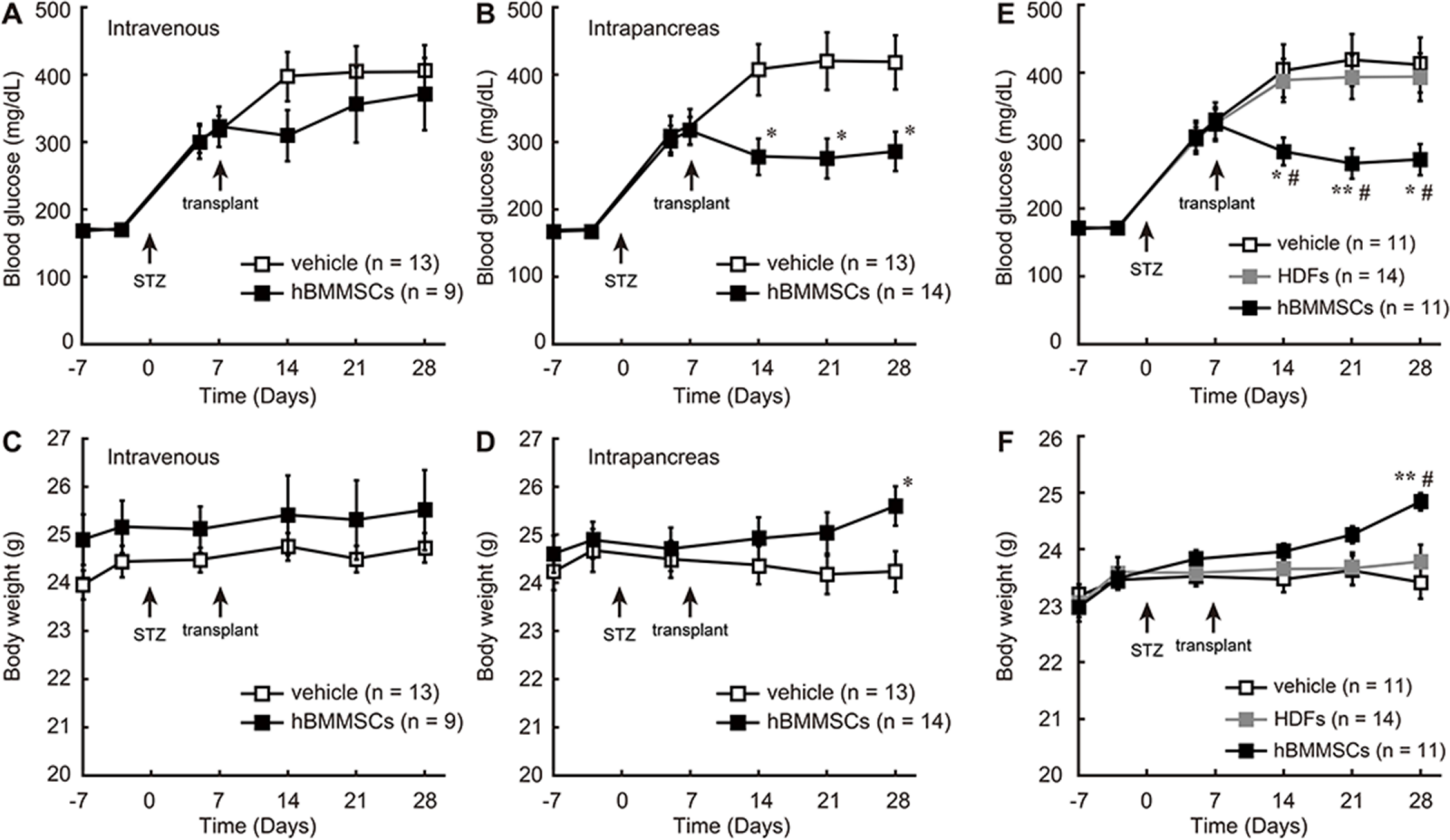
Effects of hBMMSC injection on blood glucose and body weight of STZ-induced diabetic mice. Mice were injected intravenously (A, C) or intrapancreatically (B, D) with hBMMSCs or the vehicle on day 7 after STZ treatment, and their blood glucose (A, B) and body weight (C, D) were monitored until day 28. Mice were intrapancreatically injected with hBMMSCs, HDFs, or the vehicle on day 7 after STZ treatment, and their blood glucose concentration (E) and body weight (F) were monitored until day 28. Data are expressed as the mean ± SEM. *p < 0.05, **p < 0.01 (vs vehicle injection); #p < 0.05 (vs HDF injection) (Tukey post-hoc test).

The mean blood glucose concentration of mice prior to STZ treatment was 169 ± 4 mg/dL (days −7 and −3), which increased to 317 ± 12 mg/dL on day 7 after STZ injection. To evaluate the effects of hBMMSCs on blood glucose and body weight, hBMMSCs or the vehicle (HBSS) were administered to STZ-treated mice via the pancreas or jugular vein. Blood glucose levels continued to increase following intrapancreatic or intravenous injection of the vehicle, peaking at approximately 400 mg/dL (Fig 1A and 1B). Although blood glucose levels tended to decrease following intravenous injection of hBMMSCs, the concentrations did not differ significantly from those in vehicle-treated mice (Fig 1A). Intrapancreatic injection of hBMMSCs significantly reduced mean blood glucose to 278 ± 27 mg/dL on day 14. This lower level of approximately 280 mg/dL was maintained until day 28 (Fig 1B). Body weights of mice injected with hBMMSCs into the pancreatic region were significantly higher than those of mice injected with the vehicle on day 28, while an insignificant difference was observed in the body weight between mice injected with hBMMSCs into the vein and those injected with the vehicle into the vein (Fig 1C and 1D).

### Experiment 2

#### Effects of intrapancreatic injection of hBMMSCs and HDFs on blood glucose and body weight of STZ-induced type 1 diabetic mice

To determine whether the effects of hBMMSCs were not due to xenoreactions on the part of mice against human cells, we compared the effects of the vehicle, HDFs, and hBMMSCs injected into the pancreatic region of STZ-treated mice. Diabetic animals injected with hBMMSCs had significantly decreased blood glucose levels and increased body weights compared with those injected with the vehicle. However, HDF-injected animals exhibited similar blood glucose levels and body weights as vehicle-injected mice and had significantly greater blood glucose and lower body weights than hBMMSC-injected mice during the experimental periods (Fig 1E and 1F).

### Experiment 3

#### Tracing of intrapancreatically and intravenously injected hBMMSCs

**Fig 2:**
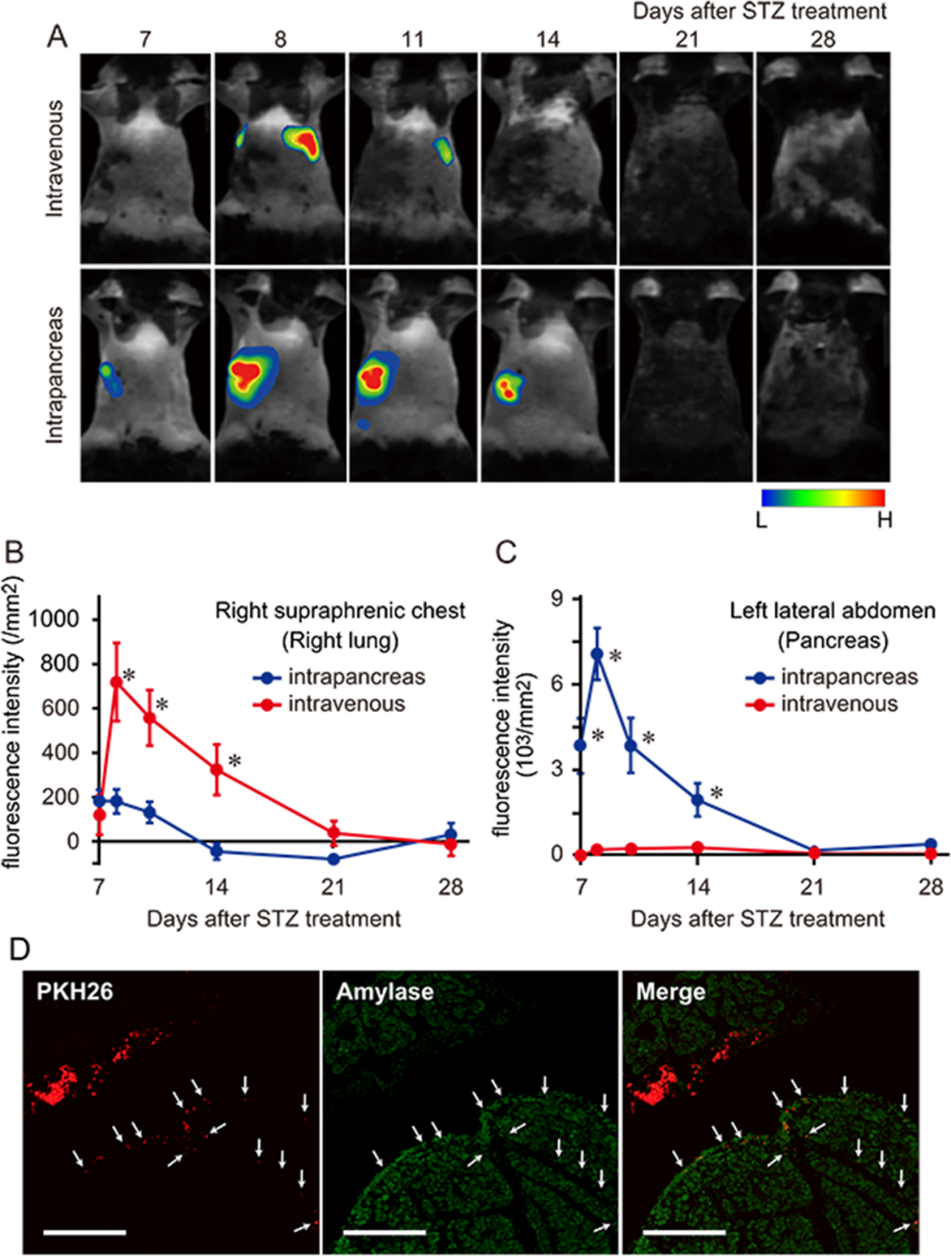
hBMMSC distribution following intrapancreatic or intravenous injection. Representative time-course images of diabetic mice injected with NIR815-labeled hBMMSCs into the jugular vein or pancreatic region (A) and semi-quantification graphs of the signal intensities in the right supraphrenic chest (B) and left lateral abdomen under the subphrenic chest (C) (n = 8 per group). Data are expressed as the mean ± SEM. *p < 0.05, **p < 0.01 (Student’s t-test). (D) The injected PKH26-labeled hBMMSCs (red and arrows) were located adjacent to the pancreas on day 14 (7 days after transplantation). Immunostaining of amylase, an exocrine pancreatic marker, is shown in green. Scale bars = 500 μm.

Previous studies have reported that intravenously injected hBMMSCs are trapped in alveolar capillaries with less distribution to peripheral tissues [18, 21, 38]. To compare the body distributions and fate of hBMMSCs delivered by intrapancreatic or intravenous injection in diabetic mice, NIR815-labeled hBMMSCs were injected via both routes, and fluorescence signals were tracked by an *in vivo* fluoroimager in a time-dependent manner. Few fluorescence signals were detected in mice injected with the vehicle into the pancreas and vein (data not shown). Following intravenous cell injection on day 7, fluorescence signals were detected mainly in the chest region. These signals clearly increased on day 8, suggesting that hBMMSCs had localized to the lungs. The fluorescence signals gradually decreased and were hard to detect within a week of cell injection, while they did not increase in other regions including the left lateral abdomen corresponding to the pancreas. However, following intrapancreatic injection, fluorescence signals were detected in the left lateral abdomen from day 7 with a dramatic increase on day 8, followed by a gradual reduction until day 21 (Fig 2A). The fluorescence intensities in the right supraphrenic chest and subphrenic left lateral abdomen were quantified in these mice. In the right supraphrenic chest corresponding to the lung, signal intensities following intravenous injection on days 8, 11, and 14 were greater than those following intrapancreatic injection. The two groups subsequently exhibited similar signal intensities in this area from day 21 (Fig 2B). Conversely, in the subphrenic left lateral abdomen corresponding to the pancreas, signal intensities following intrapancreatic injection on days 8, 11, and 14 were significantly greater than those following intravenous injection. The intensities were similar on day 21 onward and declined over time (Fig 2C).

#### Pancreatic tissue distribution of intrapancreatically injected hBMMSCs

Having confirmed the distribution of hBMMSCs in the pancreas for intrapancreatic injection, we next examined the tissue distribution of hBMMSCs after intrapancreatic injection by histochemistry using PKH26-labeled hBMMSCs. PKH26-labeled hBMMSCs were injected into the pancreatic region on day 7, and the red fluorescence signals were observed under a microscope on day 14. A cluster of red fluorescence was detected in the region just outside of the positive area for amylase, an exocrine pancreatic marker. Indeed, some red signals were also present in the narrow interlobular spaces of the exocrine pancreas. However, few red signals were detected in the pancreatic parenchyma (Fig 2D). We could not identify signals in islets (data not shown), and signals could not be sufficiently observed after day 21.

### Experiment 4

#### Effect of a second intrapancreatic injection of hBMMSCs on blood glucose and body weight

Although a single intrapancreatic injection of hBMMSCs ameliorated hyperglycemia, most injected hBMMSCs might disappear from the pancreas within 1 month. Therefore, we investigated whether a second intrapancreatic injection of hBMMSCs could further ameliorate glucose homeostasis in mice with STZ-induced diabetes mellitus.

**Fig 3:**
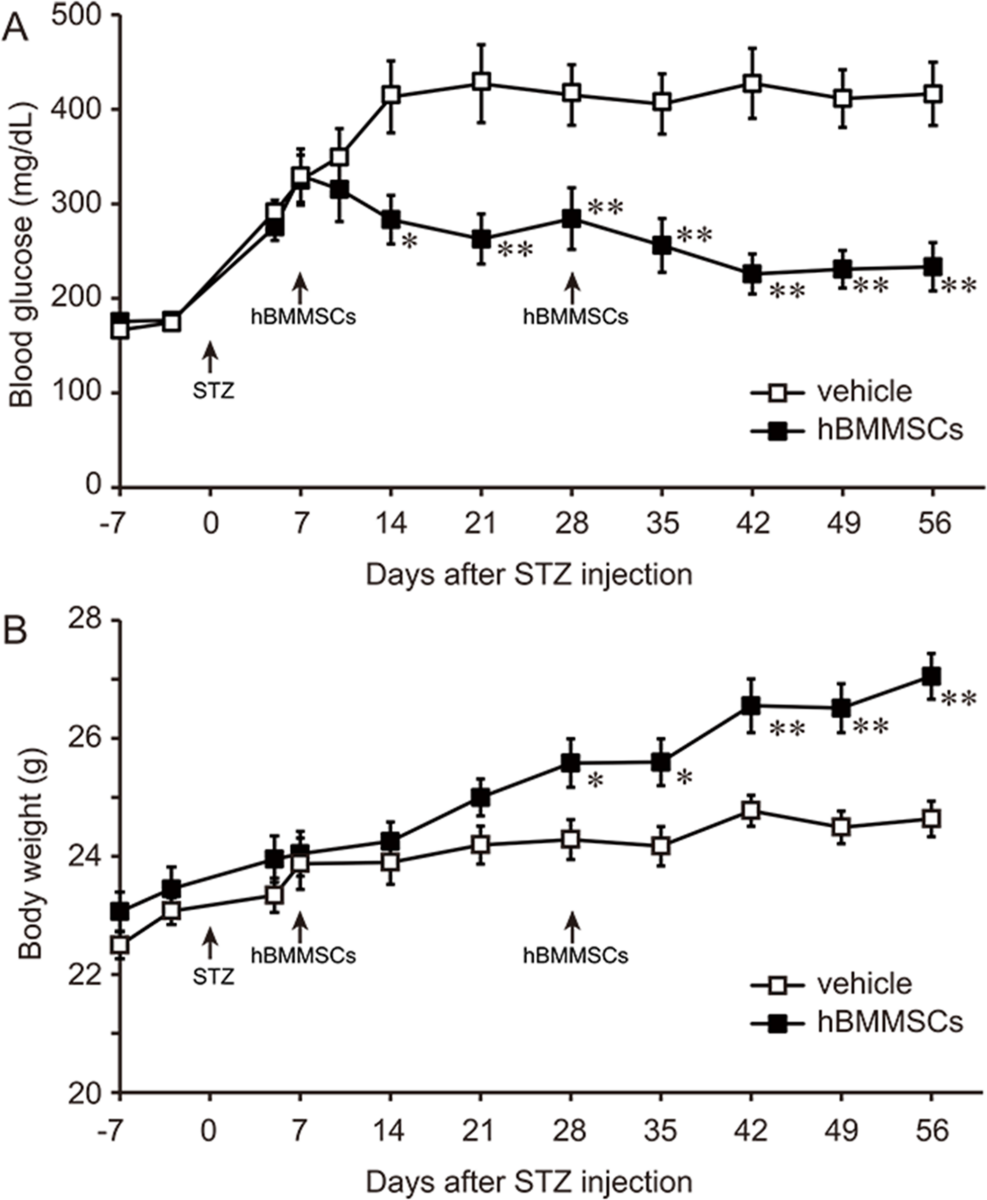
A second intrapancreatic injection of hBMMSCs lowers the blood glucose concentration and increases the body weight of STZ-induced diabetic mice. Effects of two hBMMSC injections into the pancreatic region on blood glucose (A) and body weight (B) of STZ-treated mice. The mice were injected with hBMMSCs (n = 11) or the vehicle (n = 12) on days 7 and 28, and monitored until day 56. Data are expressed as the mean ± SEM. *p < 0.05, **p < 0.01 (Student’s t-test).

A second dose of cells was injected into the pancreas on day 28, and blood glucose and body weight were monitored further until day 56. This second intrapancreatic injection of hBMMSCs on day 28 further reduced the blood glucose concentration to 233 ± 26 mg/dL on day 56, which was significantly lower than the level in vehicle-treated mice (416 ± 34 mg/dL; Fig 3A). The second injection also further increased body weight on day 56 to 27.1 ± 0.4 g, which was significantly higher than that of vehicle-injected mice (24.6 ± 0.3 g; Fig 3B).

#### Effect of a second intrapancreatic injection of hBMMSCs on plasma insulin and pancreatic histology

**Fig 4:**
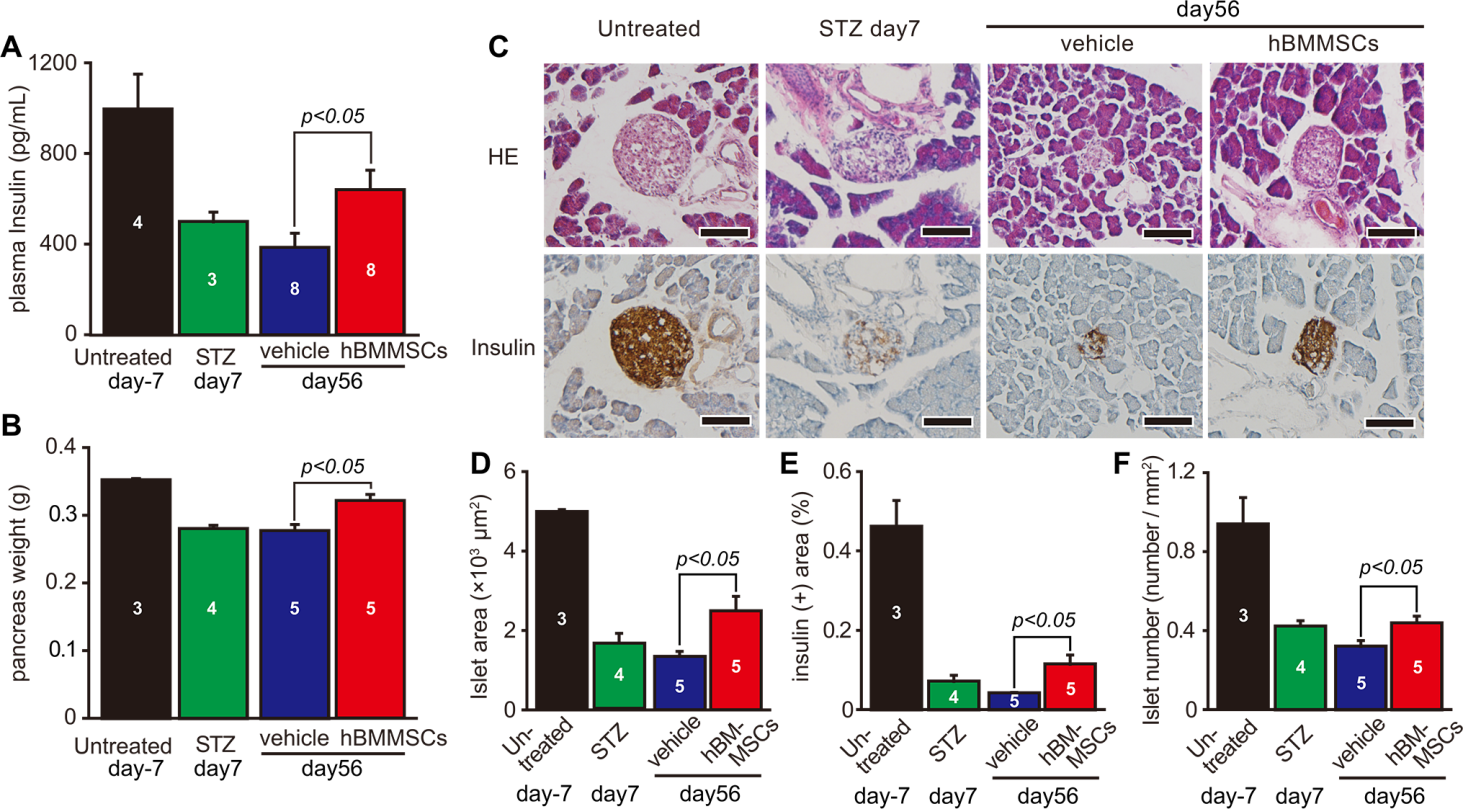
Effects of hBMMSCs on plasma insulin and pancreatic morphology on day 56. Plasma insulin levels (A), pancreas weight (B), and islet histology with H.E. staining and insulin immunostaining (C) in mice intrapancreatically injected twice with hBMMSCs or the vehicle were determined on day 56. Simultaneously, these parameters were determined in STZ-untreated control (untreated) mice on day 7 after STZ treatment (STZ day 7 or STZ). Using histological sections, the islet area (D), insulin positive (+) area (E), and islet number (F) were determined. Sample numbers are indicated in each bar. Data are expressed as the mean ± SEM and compared between vehicle- and hBMMSC-injected groups on day 56 by the Student’s t-test. Scale bars in (C) = 100 μm.

We then examined plasma insulin and pancreatic histology on day 56. Normal control mice (*n* = 4) had a mean plasma insulin concentration, measured by ELISA, of 996.5 ± 155.7 pg/mL, which was reduced to 499.7 ± 42.9 pg/mL on day 7 after STZ injection (*n* = 3). On day 56, plasma insulin concentrations in mice intrapancreatically injected with hBMMSCs (640.1 ± 85.7 pg/mL, p<0.05; *n* = 8) were significantly higher than those in mice administered with the vehicle (385.8 ± 58.0 pg/mL, *n* = 8; Fig 4A).

hBMMSC-injected mice also had a higher pancreatic weight than vehicle-injected mice (p<0.05; Fig 4B). H.E. staining of islets in normal control animals showed large round circles that were strongly positive for immunostaining of insulin. The islets in mice treated with hBMMSCs were larger than those in mice treated with the vehicle (Fig 4C). Seven days after STZ injection, the size and insulin staining intensity of the islets were decreased by day 56, and those of islets in the vehicle-treated mice had decreased further. However, in hBMMSC-treated mice, the islets had increased in size by day 56 and exhibited an increase in insulin staining intensity. On day 56, the islet area (2493 ± 362 μm^2^ vs 1343 ± 124 μm^2^, p<0.05), islet density (0.440 ± 0.033 islets/mm^2^ vs 0.322 ± 0.027 islets/mm^2^, p<0.05), and insulin-positive area (0.115 ± 0.023% vs 0.042 ± 0.002%, p<0.05) in mice intrapancreatically injected with hBMMSCs were significantly higher than those in mice injected with the vehicle (n = 5 per group; Fig 4D–4F).

### Experiment 5

#### Effect of intrapancreatic injection of hBMMSCs on the macrophage state in STZ-induced type 1 diabetic mice

It has been reported that hBMMSC transplantation has beneficial effects on several types of tissue injury via modulations of the macrophage state [27, 31, 36, 39]. We therefore tried to investigate whether intrapancreatic hBMMSC injection influenced the macrophage state.

**Fig 5:**
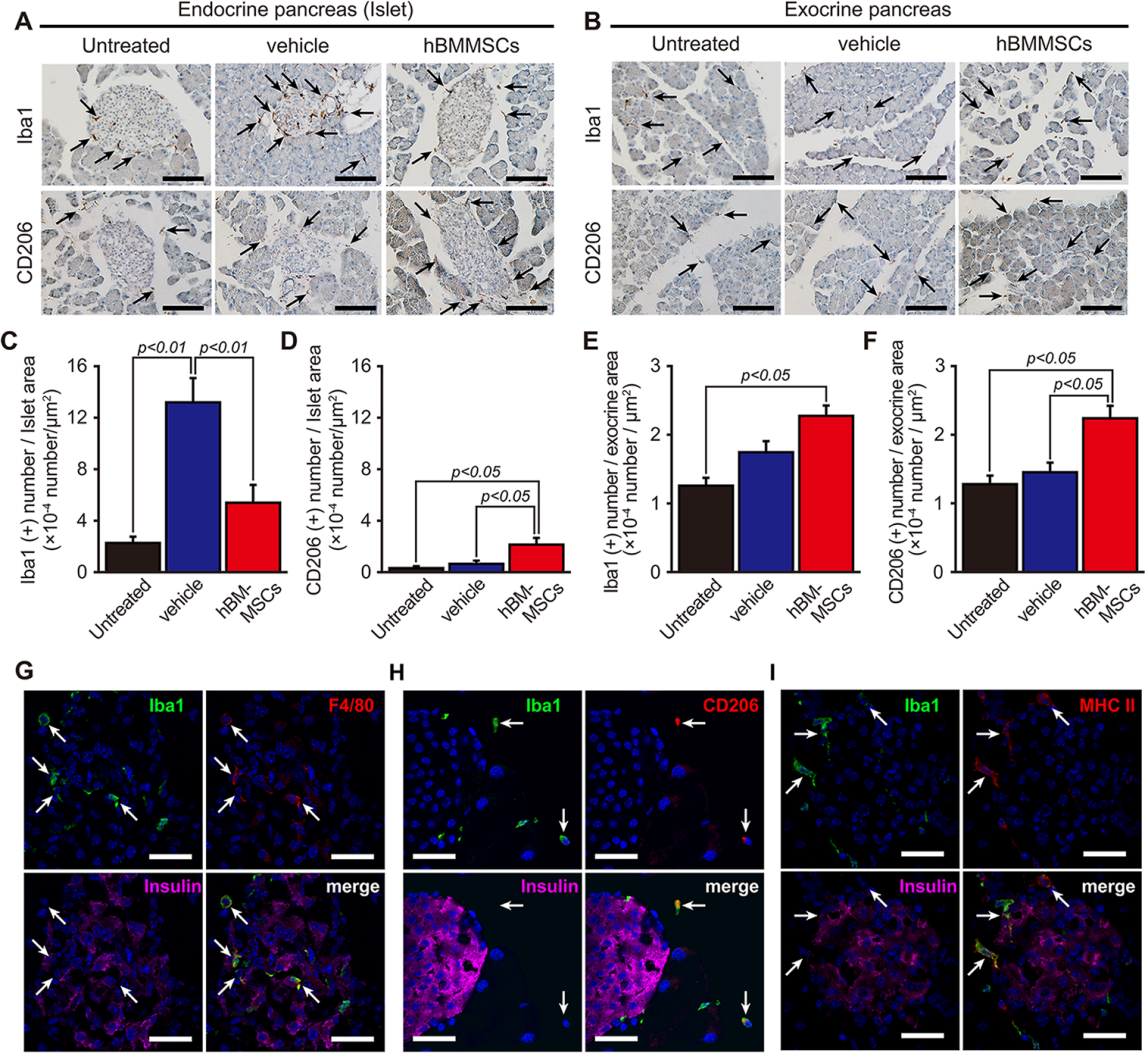
Intrapancreatic hBMMSC injection modulates the macrophage activation state. Representative images of Iba1 and CD206 immunostaining in the islets (A) and exocrine pancreas (B) of STZ-untreated control mice (untreated) and STZ-treated mice on day 28 after intrapancreatic injection of hBMMSCs or the vehicle. Scale bars = 100 μm. Semi-quantitative analysis of islets (C and D) and the exocrine pancreas (E and F) for Iba1 (C and E) and CD206 (D and F) in untreated (n = 3), vehicle-treated (n = 6), and hBMMSC-treated (n = 6) mice. Data are expressed as the mean ± SEM (Tukey post-hoc test). (G) Iba1 immunoreactions (green) were merged with F4/80 (red) immunoreactions in the islets as indicated by insulin immunoreactions (pink) in vehicle-injected mice. (H) Iba1 immunoreactions (green) were merged with CD206 immunoreactions (red) in the adjacent area of the islets (insulin immunoreactions; pink) in hBMMSC-injected mice. (I) Iba1 immunoreactions (green) were merged with MHC class II (red) in the islets and adjacent in vehicle-injected mice. DAPI staining (blue). Scale bars = 25 μm.

In STZ-untreated normal control mice, the pan-macrophage marker Iba1 was widely distributed in the islets and exocrine pancreas (Fig 5A and 5B). In vehicle-injected mice after STZ treatment, an increase of the signal intensity for Iba1 immunoreactions was observed in the islets and surrounding regions on day 28. These Iba1-positive cells exhibited macrophage-like morphologies, such as large cell bodies and cytoplasmic processes, and some of them had an activated state with large, round enlargement of the cytoplasm and short thick processes. However, intrapancreatic injection of hBMMSCs led to weak positive staining for Iba1 similar to untreated control animals (Fig 5A and 5B).

The number of Iba1-positive cells was counted in islets and exocrine pancreases. The number of Iba1-positive islets in the vehicle-injected group was approximately 6- and 4-fold higher than that in the STZ-untreated normal control group (p<0.01) and hBMMSC-injected group (p<0.01), respectively (Fig 5C). No increase of the Iba1-positive number in the vehicle-injected group was observed in the exocrine pancreas. However, the number in the hBMMSC-injected group was 2-fold higher than that in vehicle-treated mice (p<0.01; Fig 5E).

#### Intrapancreatic injection of hBMMSCs increases CD206-positive macrophages

Examination of the number of Iba1-positive cells revealed opposing results in the region of islets and the exocrine pancreas between vehicle- and hBMMSC-injected groups. We therefore performed immunostaining of CD206, a marker of M2-type macrophage activation, in these mice because we have reported that transplanted hBMMSCs increase this type of microglia/macrophage in neural damage and decrease inflammatory responses [27, 36]. A small amount of CD206-positive cells was observed in the peri-islet area and exocrine pancreas of the untreated group (Fig 5A and 5B). However, the numbers of CD206-positive cells in hBMMSC-injected mice, both in the islets and exocrine pancreas, were significantly greater than those in either the STZ-untreated control or vehicle-pancreas group (p<0.05, Fig 5D and 5F).

Pancreatic sections were then multi-stained to confirm the activation phenotype of Iba1-positive cells. Cells positive for Iba1 in the islets of vehicle-injected mice were also positive for F4/80, another pan-macrophage marker that is known to increase during inflammation (Fig 5G). Some Iba1-positive cells in mice injected with hBMMSCs were positive for CD206, suggesting an M2 phenotype outside the islets (Fig 5H), as well as MHC class II (Fig 5I).

#### Intrapancreatic injection of hBMMSCs modifies the CD40-positive cell number

**Fig 6:**
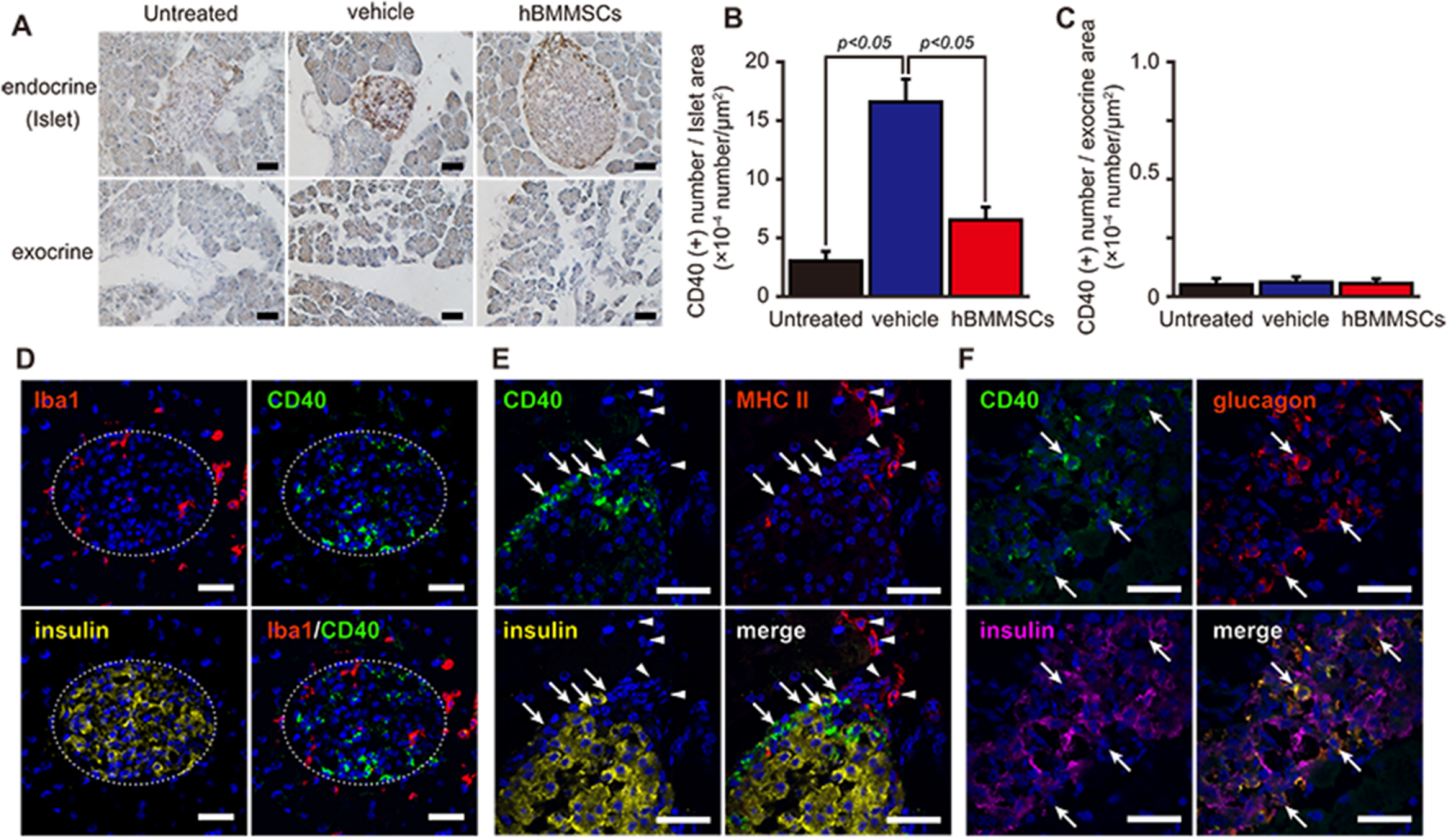
Intrapancreatic hBMMSC injection reduces CD40 immunoreactivity in islets. Representative images (A) and semi-quantification of CD40 immunostaining in the islets (B) and exocrine pancreases (C) of STZ-untreated control mice (n = 3; untreated) and mice intrapancreatically injected with the vehicle or hBMMSCs (n = 6 per group). Scale bars = 50 μm. Data are expressed as the mean ± SEM. (Tukey post-hoc test). (D) CD40 immunoreactions (green) were observed inside of the islet (insulin immunostaining; yellow), indicated by a dashed circle, and were not merged with Iba1 immunoreactions (red) in vehicle-injected mice. (E) CD40 immunoreactions (green and arrow) mainly localized to the marginal area of the larger islet and were not merged with MHC class II (red and arrowhead) or insulin (yellow) in hBMMSC-injected mice. (F) CD40 immunoreactions (green) in vehicle-injected mice were found in the islet and were merged with glucagon (red) immunoreactions, indicated by arrows, but not insulin (pink) immunoreactions. DAPI staining (blue). Scale bars = 25 μm.

CD40 is a costimulatory protein found on antigen-presenting cells (APCs), including macrophages, and is essential for dendritic cell activation [40]. CD40 is also expressed by non-immune cells during inflammatory responses including those in diabetes [41–43]. Very weak and ambiguous immunoreactivity was observed in STZ-untreated normal control mice (Fig 6A). For the vehicle-injected group, the intensity and number of dark brown CD40 immunoreactions on day 28 were obviously increased in the islets, but not in the exocrine pancreases. In the hBMMSC-injected group, on the other hand, immunoreactions were also recognized in the islets. CD40 immunoreactions were high in the marginal part of large islets (Fig 6A). The number of CD40 immunoreactions in the islets in the vehicle-injected group (16.6 ± 1.9 × 10^−4^ /μm^2^) was significantly greater than that in STZ-untreated normal control group (3.0 ± 0.8 × 10^−4^/μm^2^) and that in hBMMSC-pancreas group (6.5 ± 1.1 × 10^−4^/μm^2^, p<0.05) (Fig 6A and 6B). In contrast, CD40-positive reactions in exocrine pancreases were very low and not different among the groups (Fig 6A and 6C).

Multi-staining showed that CD40 immunoreactions did not colocalize with Iba-1, MHC class II, or insulin immunoreactions (Fig 6D and 6E) or with positivity for B220 or CD11c (data not shown). However, CD40 immunostaining clearly colocalized with glucagon immunoreactions (Fig 6F).

## Discussion

Therapeutic approaches are needed to delay the progression of β-cell destruction and replace lost insulin production in patients with type 1 diabetes mellitus. Therapies based on stem cells such as MSCs have been explored as an alternative therapeutic approach for type 1 diabetes mellitus and have beneficial effects in the animal model [22, 23, 44]. The choice of delivery route may be a critical factor determining the sustainability of implanted MSCs. Therefore, in this study, we compared the effects of hBMMSCs by intrapancreatic (local) and intravenous (systemic) injections on STZ-induced diabetic mice. Intrapancreatic injection of the cells significantly lowered the blood glucose concentration and increased body weight, whereas the effects of intravenous injection on these parameters were insignificant. Because intrapancreatically injected HDFs as a xenograft control could not reproduce the beneficial effects of hBMMSCs, the effects were specific and not nonspecific xenograft responses to hBMMSCs. Intrapancreatically injected hBMMSCs showed greater retention in the pancreas than intravenously injected hBMMSCs, although most cells, even in the former group, decreased within 2 weeks of the injection. These results suggest that the effect of hBMMSCs might be closely associated with local delivery. In this study, hBMMSCs were injected into the pancreatic region by opening the abdomen. Such an invasive procedure is not suitable for human therapy. Clinically, however, pancreatic cancer has been treated by gastric fiber-guided intrapancreatic injection of chemotherapeutic agents [45, 46], which suggests that intrapancreatic transplantation of hBMMSCs could be possible in patients with type 1 diabetes mellitus. Taken together with the findings of the current study, it suggests that cell transplantation into the pancreas might be a feasible novel therapeutic strategy.

Although a single hBMMSC injection into the pancreatic region significantly decreased the blood glucose concentration in STZ-induced diabetic mice, the concentration was approximately 2-fold higher in non-diabetic mice, and most hBMMSCs had disappeared from the pancreas. Therefore, we performed a second hBMMSC injection into the pancreatic region on day 28 and found a further decrease in the blood glucose concentration to borderline diabetic levels on day 56. hBMMSCs injected into the pancreas significantly increased the body weight of the animal. Clinically, compared with healthy individuals, type 1 diabetic mellitus patients usually have lower body weight with an insulin deficiency induced by hyperglycemia and higher energy consumption of fat and muscles [47, 48]. Although we did not examine the fat pad mass or glucose tolerance, the increase in body weight might reflect an improvement of diabetic symptoms.

It has been reported that diabetic symptoms improve by suppression of immune responses [23, 49], anti-inflammation [50–54], and enhancing β-cell or insulin-secreting cell regeneration [55, 56] and angiogenesis [53, 54]. Moreover, an increase of α-cells or glucagon-secreting cells in damaged islets might be a negative factor in diabetes [57, 58]. hBMMSCs have been reported to differentiate into β-cells [22, 59], support pancreatic progenitor cell proliferation [60], suppress immune/inflammatory responses [23, 59], and increase angiogenesis [60].

Under inflammatory conditions, hBMMSCs modulate the state of macrophage-lineage cells by cell-cell communication, resulting in anti-inflammation [27, 31, 36, 37]. We have reported that local hBMMSC injection increases the macrophage anti-inflammatory M2 subtype during brain ischemia and spinal cord injury, suggesting that hBMMSCs decrease neural damage [27, 36]. We therefore confirmed the effect of hBMMSCs on the macrophage state in the STZ-induced diabetic model. We determined that intrapancreatically injected hBMMSCs influenced the macrophage number and activation state in islets and exocrine pancreases by pan-macrophage marker Iba1 and a M2 subtype marker, CD206. Mice injected with hBMMSCs had a decrease in the Iba1-positive cell number of islets, but increased CD206-positive cells in both islets and the exocrine pancreas. Iba1-positive cells in the islets of mice injected with vehicle also exhibited thick cytoplasmic processes and large cell bodies, and partially colocalized with F4/80. While F4/80 is another pan-macrophage marker, its expression increases according to macrophage M1 activation. CD206 is a subtype marker of alternatively activated M2 macrophages which is induced by IL-4, and contributes to tissue resolution and repair [35]. We have reported that transplanted hBMMSCs increase IL-4 and induce this subtype of macrophage after CNS diseases [27, 36]. The alternative activated M2 macrophages were recruited to the pancreas in experimental models to promote β-cell survival [53–55]. These findings suggest that hBMMSCs contributed to the resolution of the islets.

We also found that the signal intensity and number of CD40-positive cells were increased in islets by STZ treatment, and that this density of CD40 in islets was decreased significantly by hBMMSC injection. CD40, a glycoprotein belonging to the tumor necrosis factor receptor superfamily, is a costimulatory protein that is expressed on APCs, including dendritic cells, activated macrophages, and mature B cells, and contributes to autoimmunity [41, 49]. CD40–CD40 ligand (CD154) signaling has been found to enhance vascular inflammation [61], bowel disease [62], and nephropathy [63]. CD154 treatment increases the levels of inflammatory cytokines in cultured β-cells [64], whereas treatment with an anti-CD154 antibody decreases blood glucose and increases pancreas allograft engraftment [65, 66]. Although we initially expected to observe CD40 expression in macrophages and/or APCs, few CD40-positive cells expressed APC or macrophage markers. CD40 has been reported to be expressed by non-immune cells [41–43, 67–69]. For example, CD40 is expressed in retinal endothelial cells and Müller cells [70], as well as in islet β-cells [43] during hyperglycemia. In this study, we observed CD40 in the peripheral area of the islets, mostly in glucagon-positive α-cells. Recently, increases in glucagon and/or the number of α-cells were reported to be essential to elevate blood glucose levels [71]. Blood glucose does not increase in mice with α-cell degeneration caused by conditional deletion of the X-linked aristaless-related homeobox gene, even when β-cells are disrupted by STZ injection [57]. Moreover, normal blood glucose levels are observed in glucagon receptor gene-deficient mice [58]. We found that the CD40-positive area, which overlapped with α-cells, was increased after STZ treatment and reduced by intrapancreatic injection of hBMMSCs. Taken together, these findings suggest that the reduction in CD40, overlapped with glucagon-positive reactions caused by hBMMSC injection might be associated with glucose homeostasis. Further studies are needed to determine the role of CD40 in α-cells and the mechanism by which hBMMSCs suppress glucagon levels and/or reduce the α-cell number.

In the present study, we did not show that hBMMSCs increased regeneration of the islets. It is not likely that transplanted hBMMSCs actively differentiated into β-cells because we could not find PKH26-labeled hBMMSCs in the islets. However, on day 56 of experiment 4, hBMMSC-injected animals had an improved plasma insulin level, pancreas weight, and histomorphological level of islets including the number, size, and insulin immunoreactions compared with vehicle-injected mice. The plasma insulin level, pancreas weight, and histomorphological level of islets in hBMMSC-injected animals on day 56 also tended to be greater than those in animals on day 7 before STZ injection, but the difference was insignificant. Further studies need to demonstrate the role of hBMMSCs, including pancreatic degeneration and/or regeneration in the future.

A future study should demonstrate a direct causal link between the modulation of pancreatic inflammation/immune responses including macrophage polarization and/or CD40-positive glucagon cells and the glucose-lowering effect of intrapancreatic hBMMSC injection. Because the STZ-induced diabetes model might not model human type 1 diabetes, we would also like to evaluate the effects of intrapancreatic hBMMSC injection in other animal models of type 1 diabetes such as non-obese diabetic mice, and the interactions between hBMMSCs and immune cells *in vitro*.

## Conclusions

In the present study, we demonstrated that intrapancreatic injection of hBMMSCs prevents hyperglycemia and restores body weight compared with intravenous injection into STZ-induced diabetic mice. Although the injected hBMMSCs disappeared from the pancreas within a month, the hBMMSCs improved plasma insulin levels and the islet morphohistology. Moreover, we confirmed that mice injected with hBMMSCs into the pancreas had a modulated macrophage number and activation state, and the decrease in the number of CD40-positive cells overlapped with glucagon in islets. Taken together, these findings suggest that intrapancreatic hBMMSCs injection might be an alternative therapeutic approach to restore β-cell functions in type 1 diabetes mellitus.

## Supporting information

S1 Fig Specificity of primary antibodies. Representative images of amylase immunostaining in the exocrine pancreas and glucagon in the islet of STZ-untreated control mice and Iba1 immunostaining in the islets of STZ-treated mice on day 7 compared with Rabbit IgG and the absence of primary antibodies (1st antibodies free). Representative images of F4/80, CD206, and MHCⅡ immunostaining in the islet of STZ-treated mice on day 7 compared with Rat IgG and 1st antibodies free. Representative images of CD40 immunostaining in the islet of STZ-treated mice on day 7 compared with Armenian hamster IgM and 1st antibodies free. Representative images of insulin immunostaining in the islet of STZ-untreated control mice compared with Guinea pig IgG and 1st antibodies free. Scale bar is 200 μm.
(TIF)

S2 Fig Determination of the number of sections for histological evaluation of pancreases. Pancreatic sections from non-diabetic mice were collected at intervals of 200 μm (nine sections). The sections were stained with hematoxylin-eosin and an antibody against insulin. The islet number was determined in the sections at 200 μm (nine sections). 400 μm (five sections), and 800 μm (three sections) intervals. The islet number of per unit area in each analysis and the calculated area under the curve (AUC). The results suggested that analysis of three sections at 800 μm intervals was appropriate in this study for histological evaluation.
(TIF)

## References

[1] Barton FB, Rickels MR, Alejandro R, Hering BJ, Wease S, Naziruddin B, et al Improvement in outcomes of clinical islet transplantation: 1999–2010. Diabetes Care. 2012;35(7):1436–45. doi: 10.2337/dc12-0063 ; PubMed Central PMCID: PMCPMC3379615. 22723582 10.2337/dc12-0063 PMC3379615

[2] Rickels MR. Recovery of endocrine function after islet and pancreas transplantation. Curr Diab Rep. 2012;12(5):587–96. doi: 10.1007/s11892-012-0294-3 ; PubMed Central PMCID: PMCPMC3432697. 22763730 10.1007/s11892-012-0294-3 PMC3432697

[3] Niederhaus SV, Kaufman DB, Odorico JS. Induction therapy in pancreas transplantation. Transpl Int. 2013;26(7):704–14. doi: 10.1111/tri.12122 . 23672537 10.1111/tri.12122

[4] Vendrame F, Hopfner YY, Diamantopoulos S, Virdi SK, Allende G, Snowhite IV, et al Risk Factors for Type 1 Diabetes Recurrence in Immunosuppressed Recipients of Simultaneous Pancreas-Kidney Transplants. Am J Transplant. 2016;16(1):235–45. doi: 10.1111/ajt.13426 . 26317167 10.1111/ajt.13426 PMC5053280

[5] Qi M, Kinzer K, Danielson KK, Martellotto J, Barbaro B, Wang Y, et al Five-year follow-up of patients with type 1 diabetes transplanted with allogeneic islets: the UIC experience. Acta Diabetol. 2014;51(5):833–43. doi: 10.1007/s00592-014-0627-6 ; PubMed Central PMCID: PMCPMC4801517. 25034311 10.1007/s00592-014-0627-6 PMC4801517

[6] Godfrey KJ, Mathew B, Bulman JC, Shah O, Clement S, Gallicano GI. Stem cell-based treatments for Type 1 diabetes mellitus: bone marrow, embryonic, hepatic, pancreatic and induced pluripotent stem cells. Diabet Med. 2012;29(1):14–23. doi: 10.1111/j.1464-5491.2011.03433.x . 21883442 10.1111/j.1464-5491.2011.03433.x

[7] Kern S, Eichler H, Stoeve J, Klüter H, Bieback K. Comparative analysis of mesenchymal stem cells from bone marrow, umbilical cord blood, or adipose tissue. Stem Cells. 2006;24(5):1294–301. doi: 10.1634/stemcells.2005-0342 . 16410387 10.1634/stemcells.2005-0342

[8] Tondreau T, Lagneaux L, Dejeneffe M, Delforge A, Massy M, Mortier C, et al Isolation of BM mesenchymal stem cells by plastic adhesion or negative selection: phenotype, proliferation kinetics and differentiation potential. Cytotherapy. 2004;6(4):372–9. doi: 10.1080/14653240410004943 . 16146890 10.1080/14653240410004943

[9] Pittenger MF, Mackay AM, Beck SC, Jaiswal RK, Douglas R, Mosca JD, et al Multilineage potential of adult human mesenchymal stem cells. Science. 1999;284(5411):143–7. . 10102814 10.1126/science.284.5411.143

[10] Barry FP, Murphy JM. Mesenchymal stem cells: clinical applications and biological characterization. Int J Biochem Cell Biol. 2004;36(4):568–84. doi: 10.1016/j.biocel.2003.11.001 . 15010324 10.1016/j.biocel.2003.11.001

[11] Prockop DJ, Olson SD. Clinical trials with adult stem/progenitor cells for tissue repair: let's not overlook some essential precautions. Blood. 2007;109(8):3147–51. doi: 10.1182/blood-2006-03-013433 ; PubMed Central PMCID: PMCPMC1852233. 17170129 10.1182/blood-2006-03-013433 PMC1852233

[12] Uccelli A, Prockop DJ. Why should mesenchymal stem cells (MSCs) cure autoimmune diseases? Curr Opin Immunol. 2010;22(6):768–74. doi: 10.1016/j.coi.2010.10.012 . 21093239 10.1016/j.coi.2010.10.012

[13] Bianco P, Robey PG, Simmons PJ. Mesenchymal stem cells: revisiting history, concepts, and assays. Cell Stem Cell. 2008;2(4):313–9. doi: 10.1016/j.stem.2008.03.002 ; PubMed Central PMCID: PMCPMC2613570. 18397751 10.1016/j.stem.2008.03.002 PMC2613570

[14] Dmitrieva RI, Minullina IR, Bilibina AA, Tarasova OV, Anisimov SV, Zaritskey AY. Bone marrow- and subcutaneous adipose tissue-derived mesenchymal stem cells: differences and similarities. Cell Cycle. 2012;11(2):377–83. doi: 10.4161/cc.11.2.18858 . 22189711 10.4161/cc.11.2.18858

[15] Carlsson PO, Schwarcz E, Korsgren O, Le Blanc K. Preserved β-cell function in type 1 diabetes by mesenchymal stromal cells. Diabetes. 2015;64(2):587–92. doi: 10.2337/db14-0656 . 25204974 10.2337/db14-0656

[16] Leibacher J, Henschler R. Biodistribution, migration and homing of systemically applied mesenchymal stem/stromal cells. Stem Cell Res Ther. 2016;7:7 doi: 10.1186/s13287-015-0271-2 ; PubMed Central PMCID: PMCPMC4709937. 26753925 10.1186/s13287-015-0271-2 PMC4709937

[17] Gao J, Dennis JE, Muzic RF, Lundberg M, Caplan AI. The dynamic in vivo distribution of bone marrow-derived mesenchymal stem cells after infusion. Cells Tissues Organs. 2001;169(1):12–20. doi: 47856 . 11340257 10.1159/000047856

[18] Lee RH, Pulin AA, Seo MJ, Kota DJ, Ylostalo J, Larson BL, et al Intravenous hMSCs improve myocardial infarction in mice because cells embolized in lung are activated to secrete the anti-inflammatory protein TSG-6. Cell Stem Cell. 2009;5(1):54–63. doi: 10.1016/j.stem.2009.05.003 ; PubMed Central PMCID: PMCPMC4154377. 19570514 10.1016/j.stem.2009.05.003 PMC4154377

[19] Suzuki T, Iyoda M, Shibata T, Ohtaki H, Matsumoto K, Shindo-Hirai Y, et al Therapeutic effects of human mesenchymal stem cells in Wistar-Kyoto rats with anti-glomerular basement membrane glomerulonephritis. PLoS One. 2013;8(6):e67475 doi: 10.1371/journal.pone.0067475 ; PubMed Central PMCID: PMCPMC3691173. 23826305 10.1371/journal.pone.0067475 PMC3691173

[20] Yang DY, Sheu ML, Su HL, Cheng FC, Chen YJ, Chen CJ, et al Dual regeneration of muscle and nerve by intravenous administration of human amniotic fluid-derived mesenchymal stem cells regulated by stromal cell-derived factor-1α in a sciatic nerve injury model. J Neurosurg. 2012;116(6):1357–67. doi: 10.3171/2012.2.JNS111360 . 22503125 10.3171/2012.2.JNS111360

[21] Furlani D, Ugurlucan M, Ong L, Bieback K, Pittermann E, Westien I, et al Is the intravascular administration of mesenchymal stem cells safe? Mesenchymal stem cells and intravital microscopy. Microvasc Res. 2009;77(3):370–6. doi: 10.1016/j.mvr.2009.02.001 . 19249320 10.1016/j.mvr.2009.02.001

[22] Ho JH, Tseng TC, Ma WH, Ong WK, Chen YF, Chen MH, et al Multiple intravenous transplantations of mesenchymal stem cells effectively restore long-term blood glucose homeostasis by hepatic engraftment and β-cell differentiation in streptozocin-induced diabetic mice. Cell Transplant. 2012;21(5):997–1009. doi: 10.3727/096368911X603611 . 22004871 10.3727/096368911X603611

[23] Kota DJ, Wiggins LL, Yoon N, Lee RH. TSG-6 produced by hMSCs delays the onset of autoimmune diabetes by suppressing Th1 development and enhancing tolerogenicity. Diabetes. 2013;62(6):2048–58. doi: 10.2337/db12-0931 ; PubMed Central PMCID: PMCPMC3661629. 23349496 10.2337/db12-0931 PMC3661629

[24] Lundberg J, Södersten E, Sundström E, Le Blanc K, Andersson T, Hermanson O, et al Targeted intra-arterial transplantation of stem cells to the injured CNS is more effective than intravenous administration: engraftment is dependent on cell type and adhesion molecule expression. Cell Transplant. 2012;21(1):333–43. doi: 10.3727/096368911X576036 . 21669035 10.3727/096368911X576036

[25] Zhang X, Yamaoka K, Sonomoto K, Kaneko H, Satake M, Yamamoto Y, et al Local delivery of mesenchymal stem cells with poly-lactic-co-glycolic acid nano-fiber scaffold suppress arthritis in rats. PLoS One. 2014;9(12):e114621 doi: 10.1371/journal.pone.0114621 ; PubMed Central PMCID: PMCPMC4256456. 25474102 10.1371/journal.pone.0114621 PMC4256456

[26] Wang M, Liang C, Hu H, Zhou L, Xu B, Wang X, et al Intraperitoneal injection (IP), Intravenous injection (IV) or anal injection (AI)? Best way for mesenchymal stem cells transplantation for colitis. Sci Rep. 2016;6:30696 doi: 10.1038/srep30696 ; PubMed Central PMCID: PMCPMC4973258. 27488951 10.1038/srep30696 PMC4973258

[27] Ohtaki H, Ylostalo JH, Foraker JE, Robinson AP, Reger RL, Shioda S, et al Stem/progenitor cells from bone marrow decrease neuronal death in global ischemia by modulation of inflammatory/immune responses. Proc Natl Acad Sci U S A. 2008;105(38):14638–43. doi: 10.1073/pnas.0803670105 ; PubMed Central PMCID: PMCPMC2567180. 18794523 10.1073/pnas.0803670105 PMC2567180

[28] Liu S, Jiang L, Li H, Shi H, Luo H, Zhang Y, et al Mesenchymal stem cells prevent hypertrophic scar formation via inflammatory regulation when undergoing apoptosis. J Invest Dermatol. 2014;134(10):2648–57. doi: 10.1038/jid.2014.169 . 24714203 10.1038/jid.2014.169

[29] Prockop DJ, Oh JY. Mesenchymal stem/stromal cells (MSCs): role as guardians of inflammation. Mol Ther. 2012;20(1):14–20. doi: 10.1038/mt.2011.211 ; PubMed Central PMCID: PMCPMC3255583. 22008910 10.1038/mt.2011.211 PMC3255583

[30] Le Blanc K, Mougiakakos D. Multipotent mesenchymal stromal cells and the innate immune system. Nat Rev Immunol. 2012;12(5):383–96. doi: 10.1038/nri3209 . 22531326 10.1038/nri3209

[31] Németh K, Leelahavanichkul A, Yuen PS, Mayer B, Parmelee A, Doi K, et al Bone marrow stromal cells attenuate sepsis via prostaglandin E(2)-dependent reprogramming of host macrophages to increase their interleukin-10 production. Nat Med. 2009;15(1):42–9. doi: 10.1038/nm.1905 ; PubMed Central PMCID: PMCPMC2706487. 19098906 10.1038/nm.1905 PMC2706487

[32] Sujata L, Chaudhuri S. Stem cell niche, the microenvironment and immunological crosstalk. Cell Mol Immunol. 2008;5(2):107–12. doi: 10.1038/cmi.2008.13 ; PubMed Central PMCID: PMCPMC4651246. 18445340 10.1038/cmi.2008.13 PMC4651246

[33] Vallés G, Bensiamar F, Crespo L, Arruebo M, Vilaboa N, Saldaña L. Topographical cues regulate the crosstalk between MSCs and macrophages. Biomaterials. 2015;37:124–33. doi: 10.1016/j.biomaterials.2014.10.028 ; PubMed Central PMCID: PMCPMC4245715. 25453943 10.1016/j.biomaterials.2014.10.028 PMC4245715

[34] Vogel DY, Vereyken EJ, Glim JE, Heijnen PD, Moeton M, van der Valk P, et al Macrophages in inflammatory multiple sclerosis lesions have an intermediate activation status. J Neuroinflammation. 2013;10:35 doi: 10.1186/1742-2094-10-35 ; PubMed Central PMCID: PMCPMC3610294. 23452918 10.1186/1742-2094-10-35 PMC3610294

[35] Gordon S, Taylor PR. Monocyte and macrophage heterogeneity. Nat Rev Immunol. 2005;5(12):953–64. doi: 10.1038/nri1733 . 16322748 10.1038/nri1733

[36] Tsumuraya T, Ohtaki H, Song D, Sato A, Watanabe J, Hiraizumi Y, et al Human mesenchymal stem/stromal cells suppress spinal inflammation in mice with contribution of pituitary adenylate cyclase-activating polypeptide (PACAP). J Neuroinflammation. 2015;12(1):252 doi: 10.1186/s12974-015-0252-5 ; PubMed Central PMCID: PMCPMC4346126. 10.1186/s12974-015-0252-5 PMC4346126 25889720

[37] Song D, Ohtaki H, Tsumuraya T, Miyamoto K, Shibato J, Rakwal R, et al The anti-inflammatory property of human bone marrow-derived mesenchymal stem/stromal cells is preserved in late-passage cultures. J Neuroimmunol. 2013;263(1–2):55–63. doi: 10.1016/j.jneuroim.2013.07.018 . 23998421 10.1016/j.jneuroim.2013.07.018

[38] Tatsumi K, Ohashi K, Matsubara Y, Kohori A, Ohno T, Kakidachi H, et al Tissue factor triggers procoagulation in transplanted mesenchymal stem cells leading to thromboembolism. Biochem Biophys Res Commun. 2013;431(2):203–9. doi: 10.1016/j.bbrc.2012.12.134 . 23313481 10.1016/j.bbrc.2012.12.134

[39] Choi H, Lee RH, Bazhanov N, Oh JY, Prockop DJ. Anti-inflammatory protein TSG-6 secreted by activated MSCs attenuates zymosan-induced mouse peritonitis by decreasing TLR2/NF-κB signaling in resident macrophages. Blood. 2011;118(2):330–8. doi: 10.1182/blood-2010-12-327353 ; PubMed Central PMCID: PMCPMC3138686. 21551236 10.1182/blood-2010-12-327353 PMC3138686

[40] Elgueta R, Benson MJ, de Vries VC, Wasiuk A, Guo Y, Noelle RJ. Molecular mechanism and function of CD40/CD40L engagement in the immune system. Immunol Rev. 2009;229(1):152–72. doi: 10.1111/j.1600-065X.2009.00782.x ; PubMed Central PMCID: PMCPMC3826168. 19426221 10.1111/j.1600-065X.2009.00782.x PMC3826168

[41] Rizvi M, Pathak D, Freedman JE, Chakrabarti S. CD40-CD40 ligand interactions in oxidative stress, inflammation and vascular disease. Trends Mol Med. 2008;14(12):530–8. doi: 10.1016/j.molmed.2008.09.006 . 18977174 10.1016/j.molmed.2008.09.006

[42] Klein D, Barbé-Tuana F, Pugliese A, Ichii H, Garza D, Gonzalez M, et al A functional CD40 receptor is expressed in pancreatic beta cells. Diabetologia. 2005;48(2):268–76. doi: 10.1007/s00125-004-1645-7 . 15690148 10.1007/s00125-004-1645-7

[43] Klein D, Timoneri F, Ichii H, Ricordi C, Pastori RL. CD40 activation in human pancreatic islets and ductal cells. Diabetologia. 2008;51(10):1853–61. doi: 10.1007/s00125-008-1092-y . 18661119 10.1007/s00125-008-1092-y

[44] Lee RH, Seo MJ, Reger RL, Spees JL, Pulin AA, Olson SD, et al Multipotent stromal cells from human marrow home to and promote repair of pancreatic islets and renal glomeruli in diabetic NOD/scid mice. Proc Natl Acad Sci U S A. 2006;103(46):17438–43. doi: 10.1073/pnas.0608249103 ; PubMed Central PMCID: PMCPMC1634835. 17088535 10.1073/pnas.0608249103 PMC1634835

[45] Karaca C, Cizginer S, Konuk Y, Kambadakone A, Turner BG, Mino-Kenudson M, et al Feasibility of EUS-guided injection of irinotecan-loaded microspheres into the swine pancreas. Gastrointest Endosc. 2011;73(3):603–6. doi: 10.1016/j.gie.2010.11.003 . 21238959 10.1016/j.gie.2010.11.003

[46] Oh HC, Seo DW, Lee TY, Kim JY, Lee SS, Lee SK, et al New treatment for cystic tumors of the pancreas: EUS-guided ethanol lavage with paclitaxel injection. Gastrointest Endosc. 2008;67(4):636–42. doi: 10.1016/j.gie.2007.09.038 . 18262182 10.1016/j.gie.2007.09.038

[47] Jacob AN, Salinas K, Adams-Huet B, Raskin P. Potential causes of weight gain in type 1 diabetes mellitus. Diabetes Obes Metab. 2006;8(4):404–11. doi: 10.1111/j.1463-1326.2005.00515.x . 16776747 10.1111/j.1463-1326.2005.00515.x

[48] Hebert SL, Nair KS. Protein and energy metabolism in type 1 diabetes. Clin Nutr. 2010;29(1):13–7. Epub 2009/09/27. doi: 10.1016/j.clnu.2009.09.001 ; PubMed Central PMCID: PMCPMC2822109. 19788950 10.1016/j.clnu.2009.09.001 PMC2822109

[49] Vaitaitis GM, Olmstead MH, Waid DM, Carter JR, Wagner DH. A CD40-targeted peptide controls and reverses type 1 diabetes in NOD mice. Diabetologia. 2014;57(11):2366–73. doi: 10.1007/s00125-014-3342-5 ; PubMed Central PMCID: PMCPMC4183717. 25104468 10.1007/s00125-014-3342-5 PMC4183717

[50] Uno S, Imagawa A, Okita K, Sayama K, Moriwaki M, Iwahashi H, et al Macrophages and dendritic cells infiltrating islets with or without beta cells produce tumour necrosis factor-alpha in patients with recent-onset type 1 diabetes. Diabetologia. 2007;50(3):596–601. doi: 10.1007/s00125-006-0569-9 . 17221211 10.1007/s00125-006-0569-9

[51] Eguchi K, Manabe I, Oishi-Tanaka Y, Ohsugi M, Kono N, Ogata F, et al Saturated fatty acid and TLR signaling link β cell dysfunction and islet inflammation. Cell Metab. 2012;15(4):518–33. doi: 10.1016/j.cmet.2012.01.023 . 22465073 10.1016/j.cmet.2012.01.023

[52] Liu X, Turban S, Carter RN, Ahmad S, Ramage L, Webster SP, et al β-Cell-Specific Glucocorticoid Reactivation Attenuates Inflammatory β-Cell Destruction. Front Endocrinol (Lausanne). 2014;5:165 doi: 10.3389/fendo.2014.00165 ; PubMed Central PMCID: PMCPMC4196588. 25352830 10.3389/fendo.2014.00165 PMC4196588

[53] Brissova M, Aamodt K, Brahmachary P, Prasad N, Hong JY, Dai C, et al Islet microenvironment, modulated by vascular endothelial growth factor-A signaling, promotes β cell regeneration. Cell Metab. 2014;19(3):498–511. doi: 10.1016/j.cmet.2014.02.001 ; PubMed Central PMCID: PMCPMC4012856. 24561261 10.1016/j.cmet.2014.02.001 PMC4012856

[54] Tessem JS, Jensen JN, Pelli H, Dai XM, Zong XH, Stanley ER, et al Critical roles for macrophages in islet angiogenesis and maintenance during pancreatic degeneration. Diabetes. 2008;57(6):1605–17. Epub 2008/03/28. doi: 10.2337/db07-1577 ; PubMed Central PMCID: PMCPMC2575065. 18375440 10.2337/db07-1577 PMC2575065

[55] Xiao X, Gaffar I, Guo P, Wiersch J, Fischbach S, Peirish L, et al M2 macrophages promote beta-cell proliferation by up-regulation of SMAD7. Proc Natl Acad Sci U S A. 2014;111(13):E1211–20. doi: 10.1073/pnas.1321347111 ; PubMed Central PMCID: PMCPMC3977272. 24639504 10.1073/pnas.1321347111 PMC3977272

[56] Smid JK, Faulkes S, Rudnicki MA. Periostin induces pancreatic regeneration. Endocrinology. 2015;156(3):824–36. Epub 2014/12/08. doi: 10.1210/en.2014-1637 . 25485969 10.1210/en.2014-1637

[57] Hancock AS, Du A, Liu J, Miller M, May CL. Glucagon deficiency reduces hepatic glucose production and improves glucose tolerance in adult mice. Mol Endocrinol. 2010;24(8):1605–14. doi: 10.1210/me.2010-0120 ; PubMed Central PMCID: PMCPMC2940466. 20592160 10.1210/me.2010-0120 PMC2940466

[58] Lee Y, Wang MY, Du XQ, Charron MJ, Unger RH. Glucagon receptor knockout prevents insulin-deficient type 1 diabetes in mice. Diabetes. 2011;60(2):391–7. doi: 10.2337/db10-0426 ; PubMed Central PMCID: PMCPMC3028337. 21270251 10.2337/db10-0426 PMC3028337

[59] Ji AT, Chang YC, Fu YJ, Lee OK, Ho JH. Niche-dependent regulations of metabolic balance in high-fat diet-induced diabetic mice by mesenchymal stromal cells. Diabetes. 2015;64(3):926–36. doi: 10.2337/db14-1042 . 25277392 10.2337/db14-1042

[60] Milanesi A, Lee JW, Li Z, Da Sacco S, Villani V, Cervantes V, et al β-Cell regeneration mediated by human bone marrow mesenchymal stem cells. PLoS One. 2012;7(8):e42177 doi: 10.1371/journal.pone.0042177 ; PubMed Central PMCID: PMCPMC3413696. 22879915 10.1371/journal.pone.0042177 PMC3413696

[61] Greene JA, Portillo JA, Lopez Corcino Y, Subauste CS. CD40-TRAF Signaling Upregulates CX3CL1 and TNF-α in Human Aortic Endothelial Cells but Not in Retinal Endothelial Cells. PLoS One. 2015;10(12):e0144133 doi: 10.1371/journal.pone.0144133 ; PubMed Central PMCID: PMCPMC4692437. 26710229 10.1371/journal.pone.0144133 PMC4692437

[62] Senhaji N, Kojok K, Darif Y, Fadainia C, Zaid Y. The Contribution of CD40/CD40L Axis in Inflammatory Bowel Disease: An Update. Front Immunol. 2015;6:529 doi: 10.3389/fimmu.2015.00529 ; PubMed Central PMCID: PMCPMC4607859. 26528290 10.3389/fimmu.2015.00529 PMC4607859

[63] Yellin MJ, D'Agati V, Parkinson G, Han AS, Szema A, Baum D, et al Immunohistologic analysis of renal CD40 and CD40L expression in lupus nephritis and other glomerulonephritides. Arthritis Rheum. 1997;40(1):124–34. . 9008608 10.1002/art.1780400117

[64] Barbé-Tuana FM, Klein D, Ichii H, Berman DM, Coffey L, Kenyon NS, et al CD40-CD40 ligand interaction activates proinflammatory pathways in pancreatic islets. Diabetes. 2006;55(9):2437–45. doi: 10.2337/db05-1673 . 16936191 10.2337/db05-1673

[65] Kover KL, Geng Z, Hess DM, Benjamin CD, Moore WV. Anti-CD154 (CD40L) prevents recurrence of diabetes in islet isografts in the DR-BB rat. Diabetes. 2000;49(10):1666–70. . 11016450 10.2337/diabetes.49.10.1666

[66] Jung DY, Kim EY, Joo SY, Park JB, Moon C, Kim SH, et al Prolonged survival of islet allografts in mice treated with rosmarinic acid and anti-CD154 antibody. Exp Mol Med. 2008;40(1):1–10. doi: 10.3858/emm.2008.40.1.1 ; PubMed Central PMCID: PMCPMC2679315. 18305392 10.3858/emm.2008.40.1.1 PMC2679315

[67] Zirlik A, Bavendiek U, Libby P, MacFarlane L, Gerdes N, Jagielska J, et al TRAF-1, -2, -3, -5, and -6 are induced in atherosclerotic plaques and differentially mediate proinflammatory functions of CD40L in endothelial cells. Arterioscler Thromb Vasc Biol. 2007;27(5):1101–7. doi: 10.1161/ATVBAHA.107.140566 . 17332487 10.1161/ATVBAHA.107.140566

[68] Laxmanan S, Datta D, Geehan C, Briscoe DM, Pal S. CD40: a mediator of pro- and anti-inflammatory signals in renal tubular epithelial cells. J Am Soc Nephrol. 2005;16(9):2714–23. doi: 10.1681/ASN.2005010045 . 16033859 10.1681/ASN.2005010045

[69] Alabraba EB, Lai V, Boon L, Wigmore SJ, Adams DH, Afford SC. Coculture of human liver macrophages and cholangiocytes leads to CD40-dependent apoptosis and cytokine secretion. Hepatology. 2008;47(2):552–62. doi: 10.1002/hep.22011 . 17999420 10.1002/hep.22011

[70] Portillo JA, Schwartz I, Zarini S, Bapputty R, Kern TS, Gubitosi-Klug RA, et al Proinflammatory responses induced by CD40 in retinal endothelial and Müller cells are inhibited by blocking CD40-Traf2,3 or CD40-Traf6 signaling. Invest Ophthalmol Vis Sci. 2014;55(12):8590–7. doi: 10.1167/iovs.14-15340 ; PubMed Central PMCID: PMCPMC4280881. 25477319 10.1167/iovs.14-15340 PMC4280881

[71] Unger RH, Cherrington AD. Glucagonocentric restructuring of diabetes: a pathophysiologic and therapeutic makeover. J Clin Invest. 2012;122(1):4–12. doi: 10.1172/JCI60016 ; PubMed Central PMCID: PMCPMC3248306. 22214853 10.1172/JCI60016 PMC3248306

