# Supplementary figures and images for: Intrapancreatic injection of human bone marrow-derived mesenchymal stem/stromal cells alleviates hyperglycemia and modulates the macrophage state in streptozotocin-induced type 1 diabetic mice
Source: PLoS One. 2017 Oct 26;12(10):e0186637. doi: 10.1371/journal.pone.0186637 (PMC5657972; doi:10.1371/journal.pone.0186637)

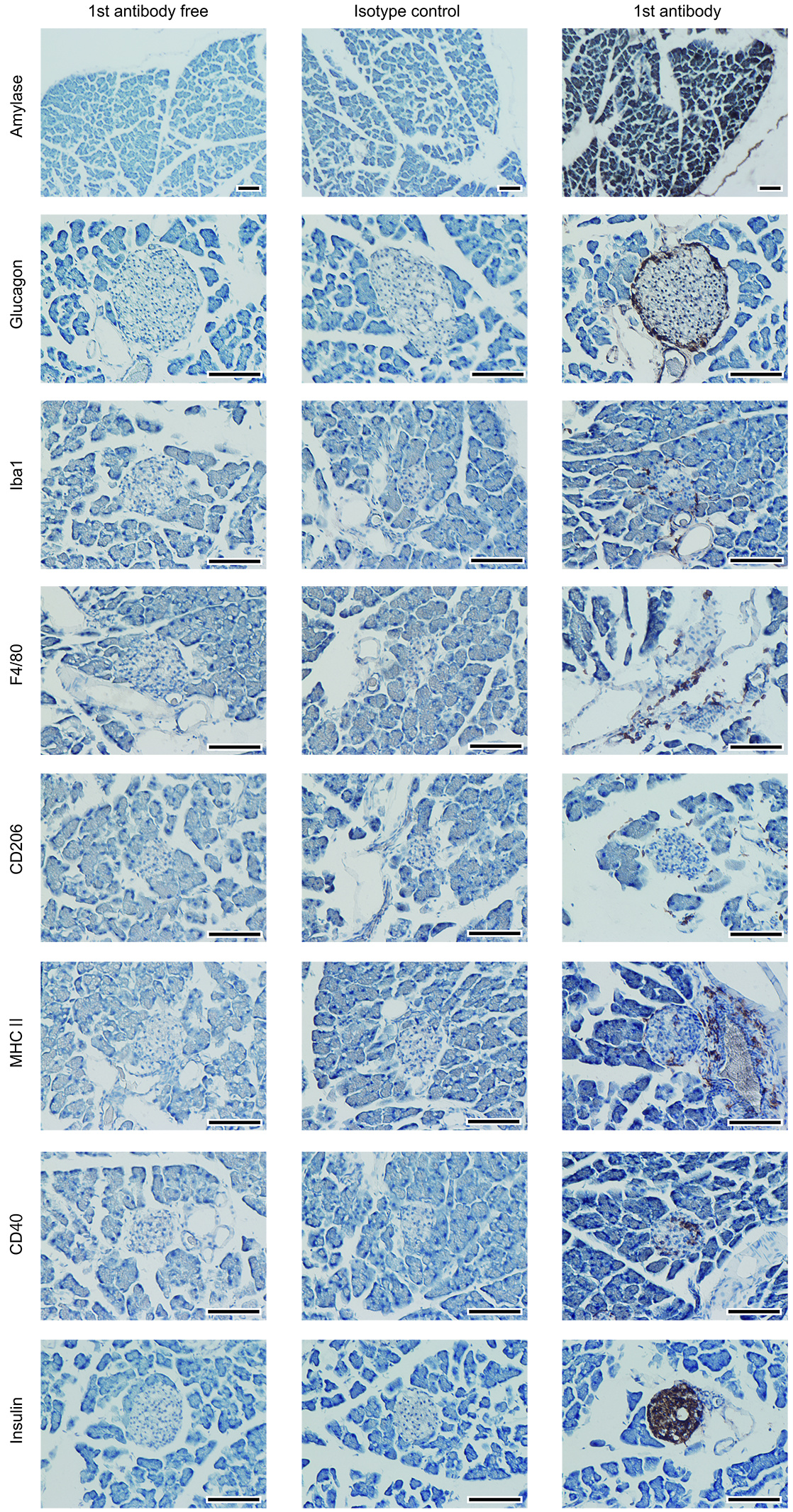

Supplement: S1 Fig — Representative images of amylase immunostaining in the exocrine pancreas and glucagon in the islet of STZ-untreated control mice and Iba1 immunostaining in the islets of STZ-treated mice on day 7 compared with Rabbit IgG and the absence of primary antibodies (1st antibodies free). Representative images of F4/80, CD206, and MHCⅡ immunostaining in the islet of STZ-treated mice on day 7 compared with Rat IgG and 1st antibodies free. Representative images of CD40 immunostaining in the islet of STZ-treated mice on day 7 compared with Armenian hamster IgM and 1st antibodies free. Representative images of insulin immunostaining in the islet of STZ-untreated control mice compared with Guinea pig IgG and 1st antibodies free. Scale bar is 200 μm. (TIF) [file pone.0186637.s001.tif]

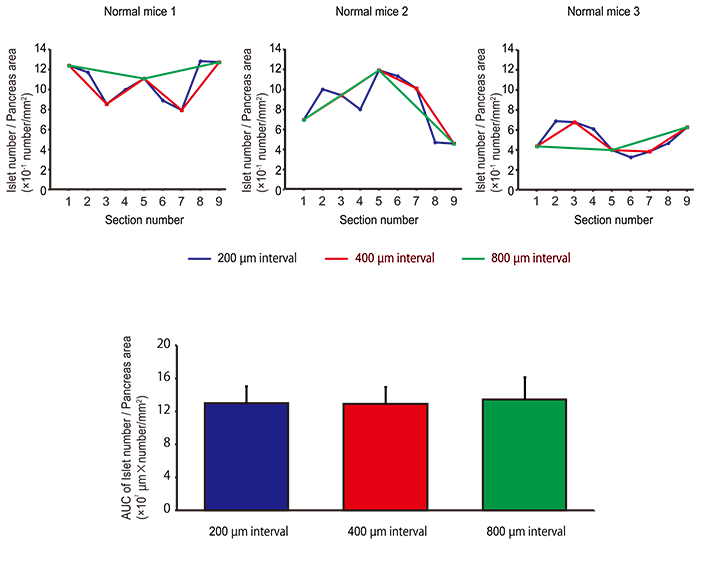

Supplement: S2 Fig — Pancreatic sections from non-diabetic mice were collected at intervals of 200 μm (nine sections). The sections were stained with hematoxylin-eosin and an antibody against insulin. The islet number was determined in the sections at 200 μm (nine sections). 400 μm (five sections), and 800 μm (three sections) intervals. The islet number of per unit area in each analysis and the calculated area under the curve (AUC). The results suggested that analysis of three sections at 800 μm intervals was appropriate in this study for histological evaluation. (TIF) [file pone.0186637.s002.tif]
